# CRISPR/Cas genome editing strategies to enhance abiotic stress tolerance in sugar crops

**DOI:** 10.3389/fgeed.2026.1942811

**Published:** 2026-10-14

**Authors:** Hong-Bo Liu, Tanweer Kumar, Farrag F. B. Abu-Ellail, Xiu-Qin Lin, Chun-Jia Li, Chao-Hua Xu, Muhammad Arif, Wajid Khan, Muhammad Ismail, Li-Ping Zhao, Ebtehag A. E. Sakr, Nur-ul Haq, Xin Lu

**Affiliations:** 1 State Key Laboratory of Tropical Crop Breeding, Sugarcane Research Institute, Yunnan Academy of Agricultural Sciences, Yunnan Key Laboratory of Sugarcane Genetic Improvement, Kaiyuan, China; 2 Agriculture Research Department Khyber, Sugar Crops Research Institute (SCRI), Mardan, Pakhtunkhwa, Pakistan; 3 Breeding and Genetics Department, Sugar Crops Research Institute (SCRI), Agricultural Research Center (ARC), Giza, Egypt; 4 Botany Department, Faculty of Women for Arts, Science, and Education, Ain Shams University, Cairo, Egypt

**Keywords:** abiotic stress, CRiSPR/Cas, genome editing, sugar beet, sugarcane, sweet sorghum

## Abstract

Sugar crops including sugarcane, sugar beets, and sweet sorghum, are fundamental for global sucrose supply and crucial feedstocks for bioethanol production and industrial bioproducts. However, yield and sucrose accumulation are significantly influenced by abiotic stresses, including drought, salinity/alkalinity, heat, chilling/freezing, and heavy metal toxicity. The polygenic complexity of stress tolerance, particularly in sugarcane with its large, polyploid, and haplotype-diverse genome, presents significant challenges for conventional breeding, rendering it slow and imprecise. This review combines advances in genome-editing technologies, including CRISPR/Cas systems (Cas9 and Cas12a), along with transcriptional techniques (CRISPRa/i), base editors, and prime editors. These tools, combined with the progress in reference genomes, haplotype-resolved assemblies, and pangenomes, have facilitated precise gene discovery, genomic selection, and targeted editing. CRISPR-based approaches offer promising avenues for enhancing key stress-responsive pathways governing oxidative homeostasis, membrane integrity, osmotic regulation, hormone signaling, and ion-homeostasis in sugar crops. Prominent candidate targets include transcription factors (TF), signaling components, raffinose biosynthesis genes implicated in freezing tolerance, regulators of salt and alkaline stress, and determinants of heavy metal tolerance and sequestration. Translational challenges, such as off-target effects, low transformation efficiencies, and editing multiple homologues or haplotypes, are being addressed such as DNA-free ribonucleoprotein (RNP) delivery, high-throughput screening, haplotype-specific target design, and haplotype-aware guide RNA engineering based on unique genetic variation sites across different gene haplotypes. Collectively, the integration of advanced CRISPR toolkits with enhanced genomic information resources and phenotyping capabilities holds significant potential for accelerating the development of precision-bred, multi-stress-resilient sugar crops that sustain yield, quality, and industrial value under abiotic stress conditions.

## 1 Introduction

Sugar crops, such as sugarcane (*Saccharum* hybrid spp.), sugar beet (*Beta vulgaris* var. *saccharifera*), and sweet sorghum (*Sorghum bicolor* (L.) Moench) are integral to global agriculture and serve as major sources of sucrose, bioenergy, and various industrial products. A defining attribute of these crops is the accumulation of high sucrose in specialized sink tissues (Kumar et al., 2014; 2024; Pandey et al., 2025). However, the productivity and sustainability of these sugar crops face significant challenges owing to abiotic stresses, including drought, salinity, extreme temperatures (both heat and cold), and heavy metal toxicity. Sugarcane produces approximately 80% of the world’s sugar and is a cornerstone of bioethanol and bioproducts, particularly in tropical and subtropical regions (Pandey et al., 2025; Liu et al., 2026). Sugar beets, mainly grown in temperate regions, are distinguished by tuberous roots and higher sugar content (Nap et al., 2025). Although sugar beet cultivation incurs higher costs and industrial use offers substantial potential for achieving higher ethanol yields than other feedstocks. To boost sugar beet productivity and ensure quality, it is vital to enhance abiotic and biotic stresses, such as disease resistance, high yield, and sugar content (Abu-Ellail et al., 2024; 2025; Nap et al., 2025). Sweet sorghum is a bioenergy and bioethanol crop known for its wide adaptability to harsh environments. Its minimal water and fertilizer requirements render it well-suited for cultivation on marginal lands, providing a sustainable alternative to maize crops. However, chilling stress constrains the adaptability of crops to cooler climates (Appiah-Nkansah et al., 2019; Yang J. et al., 2025). Genome-editing technologies can further augment this potential by enhancing cold tolerance and reducing agricultural inputs (Maulana and Tesso, 2013). These advancements could facilitate the production of value-added products, thereby broadening the commercial applications of sweet sorghum (Hossain et al., 2022; Ameen et al., 2024).

As climate change intensifies abiotic stress, enhancing stress resilience while maintaining or increasing sucrose accumulation is a critical challenge for sugar crops. Traditional breeding faces significant difficulties due to the polygenic and polyploid nature of the sugarcane genome (∼10 Gb). Numerous sequencing initiatives have targeted both parental species and hybrid genotypes. Notably, haplotype separation and assembly produced high-quality haplotype-resolved assemblies and graph pangenomes for sugarcane. These resources enable gene discovery, genomic selection, and precision editing, thereby overcoming key breeding bottlenecks. Advances in genomic resources for sugar beet and sweet sorghum likewise create new opportunities for precision breeding. These genomic resources summarized in (Supplementary Table S1) have been instrumental in advancing sugar crop improvement. For sugarcane, haplotype-resolved assemblies and graph pangenomes have enabled the identification of stress-responsive gene haplotypes for CRISPR targeting. For sugar beet, reference genomes and pangenome resources have facilitated the discovery of genes associated with salt and cold tolerance. For sweet sorghum, the telomere-to-telomere genome assembly provides a foundation for precise gRNA design. Collectively these genomic resources directly support the CRISPR-based strategies discussed in this review by enabling haplotype-aware gRNA design and functional validation of target genes. Genome editing using CRISPR/Cas systems has revolutionized plant biotechnology by enabling precise and efficient genetic modification. Tools for genome editing, including zinc-finger nucleases (ZFNs), transcription activator-like effector nucleases (TALENs), and CRISPR/Cas systems (Figure 1A), provide enhanced simplicity and scalability via RNA-guided DNA recognition. These technologies have broadened the scope for manipulating complex traits in sugar crops, such as abiotic stress tolerance (Zhang et al., 2020; Kumar et al., 2024; Pacesa et al., 2024). The application of CRISPR/Cas technology allows for the identification and editing of genes related to drought, salinity, chilling, and heavy metal tolerance, thereby enabling sugar crops to endure adverse environmental conditions while maintaining productivity.

**FIGURE 1:**
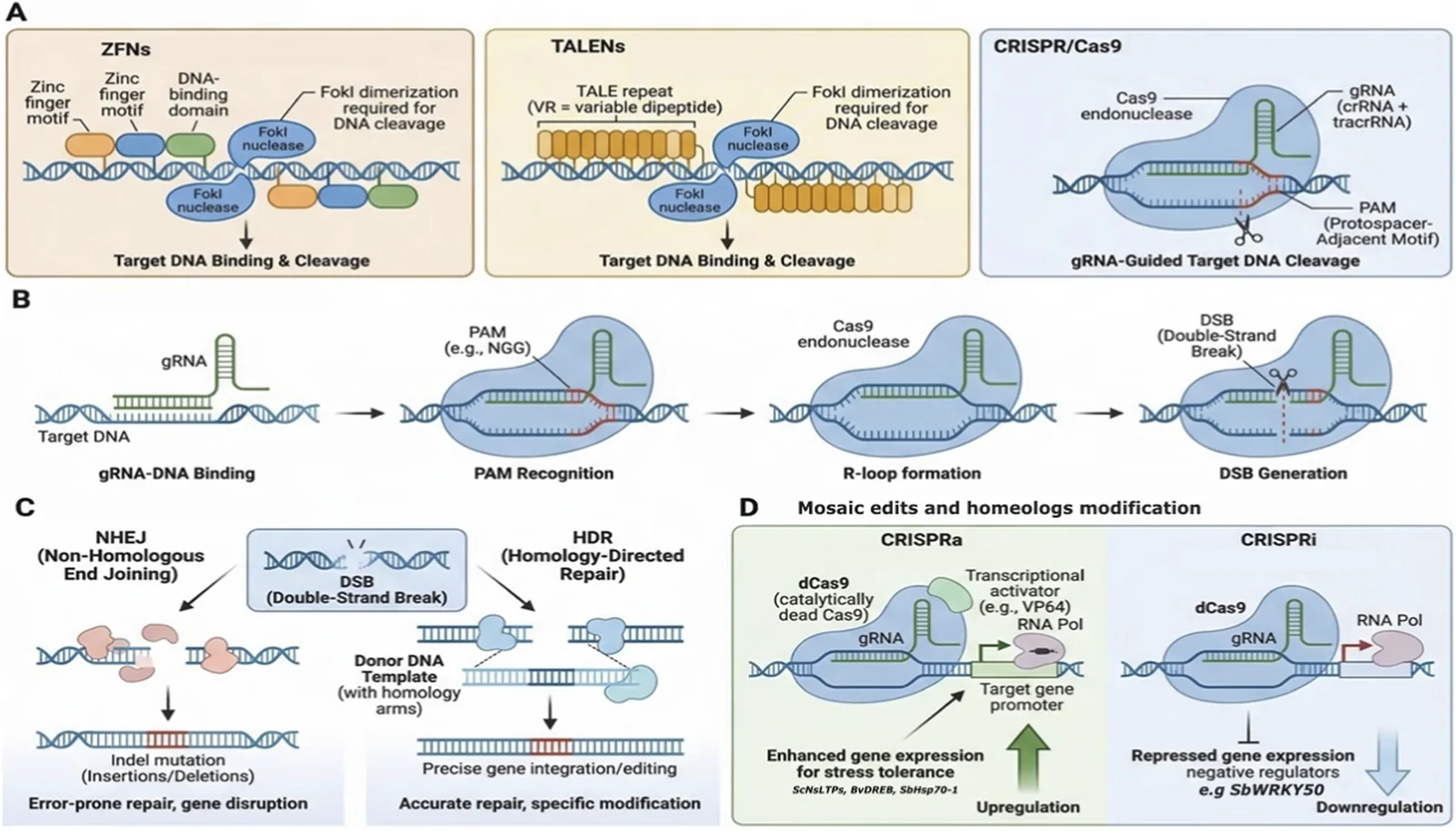
Overview of genome editing tools and CRISPR-based applications for abiotic stress tolerance in sugar crops. **(A)** Three nuclease-based editing platforms: Zinc finger nucleases (ZFNs) bind target DNA, and FokI nuclease dimerization induces site-specific double-strand breaks (DSBs). TALENs: TALE repeats (with variable dipeptides) confer DNA specificity, and FokI dimerization is required for cleavage. CRISPR/Cas9: A guide RNA (gRNA) directs the Cas9 endonuclease to a PAM-adjacent target sequence, triggering DSB formation. **(B)** CRISPR/Cas9 cleavage mechanism in sugar crops: Cas9-mediated DNA cleavage: gRNA hybridizes to target DNA. Cas9 recognizes the PAM motif. The R-loop stabilizes the Cas9-gRNA-DNA complex. Cas9 generates a DSB upstream of the PAM. **(C)** DSB repair pathways in polyploid sugar crops: Two pathways resolve DSBs: NHEJ: Error-prone ligation of broken ends generates insertions/deletions (indels) for gene disruption. HDR: High-fidelity repair using a homologous donor template, enabling precise gene insertion or modification. **(D)** CRISPRa/i for transcriptional control of abiotic stress responses in sugar crops. dCas9-based systems modulate gene expression without DNA cleavage: CRISPRa: dCas9 fused to transcriptional activators (e.g., VP64) upregulates stress tolerance genes (e.g., Hsp70 and *BVDREB* genes). CRISPRi: dCas9 blocks RNA polymerase, repressing negative regulators of stress resilience (e.g., sweet sorghum SbWRKY50 for salt tolerance). SciDraw. (2026). SciDraw AI: Scientific illustration generator. Retrieved from https://scidraw.com/aidrawing; accessed on June 20, 2026.

The integration of CRISPR/Cas tools into sugar crop breeding programs offers unprecedented opportunities to develop resilient varieties that can withstand various abiotic stressors. Genome editing offers a promising strategy to accelerate the development of stress-resilient sugar crops and support sustainable agriculture under abiotic stress.

## 2 Overview of Genome editing

CRISPR has emerged as a powerful tool for enhancing abiotic tolerance. Genome editing improves drought, salinity, and heat tolerance by targeting genes, such as aquaporins, heat shock proteins, and ion transporters (Nagaraju et al., 2019). Previous studies have demonstrated that the overexpression of specific genes and transcription factors enhances stress tolerance by augmenting the accumulation of signaling molecules (Zhang et al., 2020; Kumar et al., 2024; Pacesa et al., 2024; Pandey et al., 2025). Key TF’s families that regulate downstream genes involved in osmo-protection and stomatal regulation, such as the WRKY gene family, specific transcription factors, and the NAC family, no apical meristem (NAM), and dehydration-responsive element binding (DREB). CRISPR-based technologies, including CRISPRa and CRISPRi, offer valuable tools for regulating stress tolerance (Figures 1A–D), and genes in sugar crops (Osakabe et al., 2016; Kumar et al., 2018; 2024). Specifically, targeting drought-responsive genes using CRISPR/Cas12a holds promise for enhancing water retention and stress tolerance. CRISPR/Cas systems provide a practical approach to breeding resilient crops capable of overcoming both biotic and abiotic challenges and the development of sugar crop varieties that withstand increasing abiotic stresses induced by climate change (Yolcu et al., 2021). Genome editing encompasses genetic modification that enables precise manipulation of DNA through insertion, deletion, and replacement within plant genomes. Molecular tools, such as ZFNs, TALENs, and (CRISPR)/CRISPR-associated protein 9 (Cas9), significantly advanced plant genetics (Figures 1A–D). These technologies have been rapidly employed to enhance crop yield, improve nutritional quality, and increase resistance to abiotic and biotic stressors (Kumar et al., 2024; Li and Iqbal, 2024; Mall et al., 2024).

Genome editing tools utilize sequence-specific nucleases to recognize and bind to specific DNA sequences, inducing double-stranded breaks (DSBs). ZFNs are customizable DNA-cleaving proteins engineered to target and cleave specific locations. TALENs induce DSBs at designated sites, activating DNA repair response pathways that result in genome modifications. In the CRISPR-Cas9 system, the RNA-guided endonuclease Cas9 employs a guide RNA (gRNA) to recognize and bind to a specific target DNA sequence (protospacer) that is adjacent to a protospacer-adjacent motif (PAM). Upon binding, Cas9 introduces a double-strand break (DSB) at the target site (Zhang et al., 2020; Hussain et al., 2021). Specifically, the Cas9 ribonucleoprotein (RNP) complex, comprising the Cas9 endonuclease and guide RNA (gRNA), recognizes and binds to the target sequence through complementary base pairing between the gRNA and gDNA.

Typically, two mechanisms exist for DSB repair. The first mechanism, non-homologous end-joining (NHEJ), results in insertions or deletions (Indels). If these indels induce frameshift mutations (the length is not a multiple of three base pairs), can lead to loss-of-function mutations. The second mechanism, homology-directed repair (HDR), utilizes a donor DNA template with high sequence homology to the regions flanking the DSB to accurately repair the break (Figures 1B,C), potentially correcting existing mutations and enabling precise genetic editing (Hussain et al., 2021; Li and Iqbal, 2024; Mall et al., 2024). In addition to DSB-mediated editing, CRISPR systems can be adapted for transcriptional regulation without permanent DNA modification. CRISPR activation (CRISPRa) utilizes a catalytically inactive Cas9 (dCas9) fused to transcriptional activation domains, such as VP64 and EDLL, to enhance target gene expression (Figure 1D). In contrast, CRISPR interference (CRISPRi) uses catalytically inactive Cas9 (dCas9) to repress target gene transcription, either by sterically hindering transcription or by recruiting transcriptional repressor domains such as KRAB. Conversely, CRISPR activation (CRISPRa) couples dCas9 to transcriptional activators to enhance the expression of target genes. Together, CRISPRi and CRISPRa enable programmable modulation of gene expression without permanently altering the underlying DNA sequence, providing complementary strategies for dissecting and engineering stress-responsive pathways. (Ben-Tov et al., 2024).

The CRISPR/Cas system has significant advantages over ZFNs and TALENs because of its simplicity in design, cost-effectiveness, versatility, and efficiency. This approach allows the rapid introduction of guide RNAs to target nearly any DNA sequence with a nearby PAM, facilitating the simultaneous editing of multiple genes through multiplexing (Urnov et al., 2010; Hussain et al., 2021). By employing ribonucleoproteins (RNPs) for direct delivery into plant cells, the CRISPR/Cas system can mitigate the regulatory and public acceptance challenges associated with transgenic or genetically modified (GM) approaches in certain regions, particularly when RNPs are used to avoid foreign DNA integration. This system streamlines the creation of genetically edited plants and expedites development and adoption for future commercial cultivation (Antony Ceasar and Ignacimuthu, 2023). Consequently, enhances crop traits more efficiently and sustains food security in global challenges.

## 3 Enhancing abiotic stress tolerance by CRISPR/Cas

### 3.1 Abiotic stress challenges

Abiotic stresses, particularly those related to climate change, are the most critical constraints on sugar crop productivity worldwide. These stresses frequently occur concurrently under field conditions, resulting in complex stress conditions that exacerbate cellular damage through oxidative stress, membrane destabilization, protein denaturation, and metabolic imbalance (Figure 2). In sugar crops, these stresses not only diminish biomass accumulation but also disrupt sucrose synthesis, transport, and storage, ultimately affecting both yield and quality (Mall et al., 2024; Pandey et al., 2025). Unlike monogenic traits, abiotic stress tolerance is governed by intricate networks of genes involved in various physiological processes, such as signaling pathways, transcriptional regulation, osmotic adjustment, antioxidant defense response, hormone crosstalk, and metabolic reprogramming (Misra et al., 2023; Mall et al., 2024).

**FIGURE 2:**
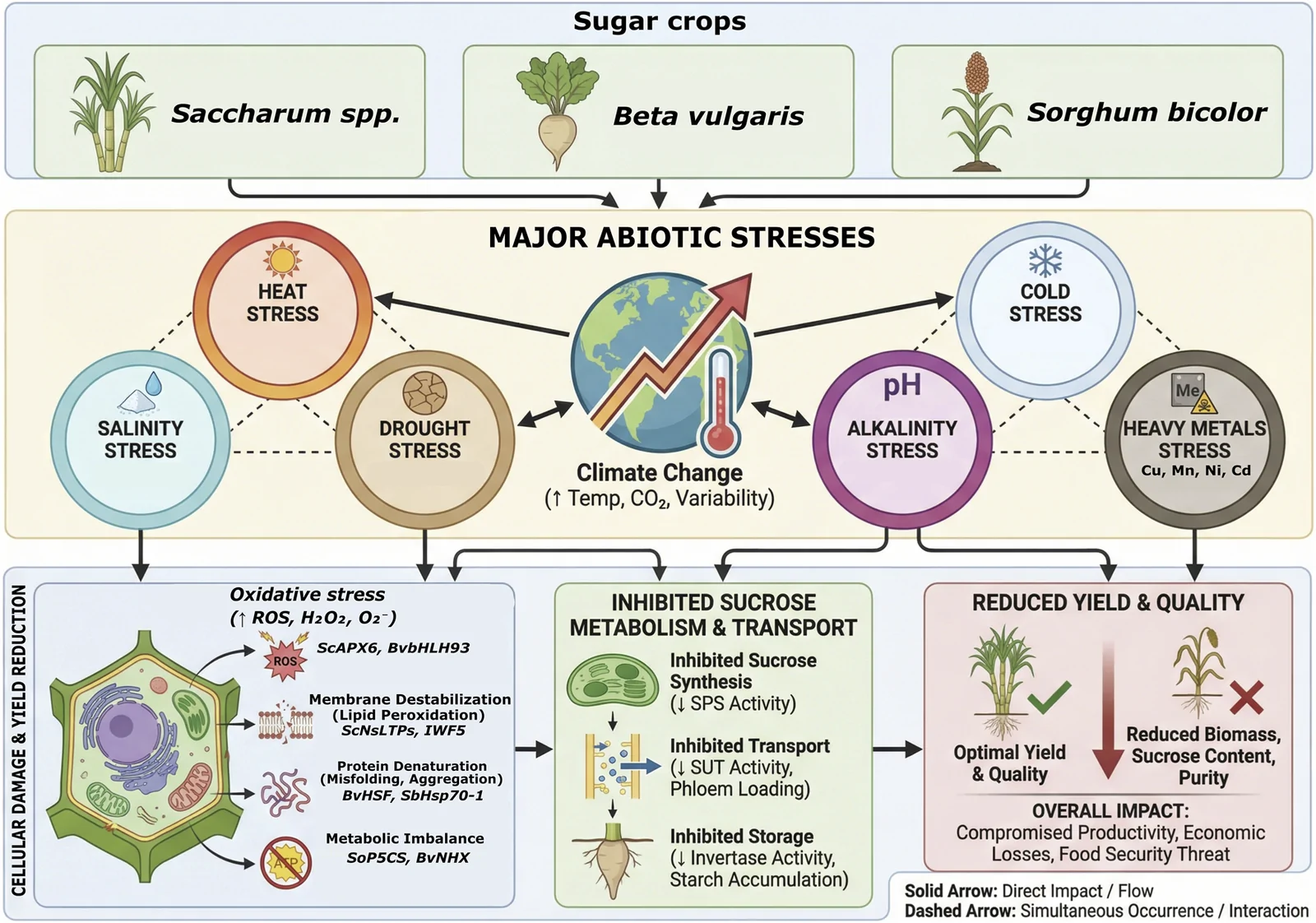
Schematic overview of climate change-driven abiotic stresses and cascading effects on cellular integrity, sucrose metabolism, and productivity in sugar crops. Major abiotic stresses induced by climate change—including heat stress, drought stress, cold stress, salinity stress, alkalinity stress, and heavy metal stress. These stresses trigger cellular damage through oxidative stress (↑ROS, H_2_O_2_, O_2_^−^) membrane destabilization, protein denaturation (misfolding, aggregation), and metabolic imbalance (inhibited enzymes, ATP depletion). Consequently, sucrose metabolism is disrupted through inhibited synthesis (↓SPS activity), transport (↓SUT activity, phloem loading), and storage (↓invertase activity). The cumulative impact results in reduced biomass, sucrose content, and purity, leading to compromised productivity, economic losses, and threats to food security. SciDraw. (2026). SciDraw AI: Scientific illustration generator. Retrieved from https://scidraw.com/aidrawing; accessed on 20 June 2026.

A primary challenge in enhancing abiotic stress tolerance in plants is the polygenic and quantitative nature of stress-responsive traits. Consequently, traditional breeding relies on phenotypic selection, which is often slow, imprecise, and inefficient, particularly in the genomic architecture of sugarcane and sugar crops.

CRISPR/Cas-based genome-editing technologies are a transformative solution that facilitates the precise and targeted modification of key stress-responsive genes and regulatory elements. By directly manipulating master/hub regulators, signaling components, and metabolic genes associated with abiotic stress tolerance, CRISPR/Cas systems offer unparalleled opportunities to elucidate stress mechanisms and engineer sugar crop varieties with enhanced resilience and productivity under environmental conditions (Misra et al., 2023; Pandey et al., 2025). Understanding the regulation of gene expression in response to drought, salinity, and temperature stress is crucial for developing resilient sugar beet varieties. Manipulating the expression of specific stress-responsive genes in sugar beets under adverse conditions will elucidate the regulatory mechanisms and facilitate the breeding of stress-tolerant sugar beet varieties. Furthermore, CRISPR/Cas12a recognizes dsDNA targets bearing a T-rich PAM, upon activation, exhibits non-specific trans-cleavage of ssDNA. For genome editing in sugar crops, Cas12a is utilized for its precise dsDNA cleavage and T-rich PAM compatibility, enabling multiplexed gene knockouts.By targeting genes involved in specific abiotic stress responses, genome editing can enhance the ability of sugar beet to withstand adverse environmental conditions (Figure 2; Table 1). For example, targeting drought-responsive genes using the CRISPR/Cas12a system can significantly improve drought tolerance in plants. These genetically modified plants demonstrated enhanced water retention capabilities, reduced wilting, and increased tolerance under drought conditions. Consequently, the utilization of CRISPR/Cas technology is vital for breeding highly resilient sugar crop varieties that can combat biotic and abiotic stress (Figures 1, 2). The application of CRISPR/Cas technology involves specific procedures for cultivating sugar crop varieties with diverse abiotic stress tolerance (Tables 1–3) respectively.

**TABLE 1:** The key target genes involved in sugarcane responses to various abiotic stresses, the genome editing approaches used to modulate these genes, their functional roles under stress, and the CRISPR/Cas tools, including CRISPRa, gene knockout, and base and prime editors, to improve stress tolerance.

Abiotic stress	Target gene	Observed phenotype	Genome editing approach	Molecular/Physiological function	CRISPR tool	References
(Category II)						
Drought	*ScD27.2*	OE: enhanced drought tolerance, reduced tillering; RNAi-silencing: increased tillering, reduced drought tolerance	Transcriptional modulation	Strigolactone biosynthetic isomerase; coordinates plant architecture and drought adaptation	CRISPRa	(Zan et al., 2023; Wu et al., 2026b)
Drought	*SoACLA-1*	Overexpression improves drought resistance in sugarcane	Gene knockout	Modulates carbon metabolic flux to enhance drought tolerance	CRISPR/Ca s9/Ca s12a; Base/Prime Editors	(Zhu et al., 2021)
Salinity, Drought	*miRNAs*	Differential expression under salinity and drought; post-transcriptional regulation of stress-responsive genes	Gene knockout	Post-transcriptional regulators of stress-responsive gene expression via small RNA machinery	CRISPR/Cas9/Cas12a; Base/Prime Editors	(Ferreira et al., 2012; Carnavale Bottino et al., 2013)
Drought, Chilling	*ScNsLTPs*	Upregulated transcript levels under drought and chilling stress	Transcriptional upregulation	Preserves cellular membrane integrity; reduces transpiration water loss	CRISPRa	(Chen et al., 2017; Hussain et al., 2018)
Drought	*ScJAZ1-1/2/3/4* *SsJAZ7-1*	Transcripts induced by JA, PEG and ABA treatments	Transcriptional upregulation	Mediates JA/ABA/PEG-induced stress signaling; bolsters drought acclimation responses	CRISPRa	(Zhou et al., 2024)
Heat	*SsHsp17.2*	Gene induced under heat and water-deficit stress	Transcriptional upregulation	Chaperone-mediated cellular protection; mitigates combined heat and water deficit stress	CRISPRa	(Tiroli and Ramos, 2007; Tiroli-Cepeda and Ramos, 2010)
Saline-Alkaline	*ScGluD2*	Transcript upregulated under salt, ABA, H_2_O_2_ and heavy-metal treatments	Transcriptional upregulation	Beta-1,3-glucanase activity; mediates stress responses to saline-alkaline soil conditions	CRISPRa	(Su et al., 2016; Wu et al., 2022)
Saline-Alkaline	*ScWRKY5*	Altered expression upon salt, SA, PEG and pathogen inoculation	Gene knockout	Modulates SA/ABA/PEG/salt stress signaling; boosts cellular ROS detoxification capacity	CRISPR/Cas9/Cas12a; Base/Prime Editors	(Wang et al., 2020)
Salinity, Drought, Oxidative stress	*ScAPX6*	Elevated transcript abundance under multiple abiotic stresses	Transcriptional up/downregulation	Ascorbate peroxidase activity; scavenges reactive oxygen species under multi-stress	CRISPRa/i	(Liu et al., 2018)
Salinity, Drought, Oxidative stress	*ScDir*	Stress-responsive transcript accumulation	Gene knockout/knock-in	Dirigent protein activity; mitigates oxidative stress-induced cellular damage	CRISPR/Cas9; Prime Editors	(Jin-long et al., 2012; Li et al., 2022b)
Drought	*ScTpx2*	Overexpression enhances drought tolerance in transgenic sugarcane	Transcriptional upregulation	Enhances photosynthesis and reduces oxidative damage; improves vascular system development for water transport	CRISPRa	(Tirabante Terrones et al., 2026)
Cold	*SsGF2.1-2.4*	Transcripts induced by cold-stress treatments	Transcriptional upregulation	Mediates cold/drought stress signaling cascades and cellular homeostasis	CRISPRa	(Zhang et al., 2024a)
Drought	*R2R3-MYB*	Drought-induced transcript changes	Transcriptional upregulation	Regulates drought-responsive gene expression; drives osmoprotective metabolite synthesis	CRISPRa	(Guo et al., 2017)
Drought, Cold, Salt, Wounding	*PScMYBAS1*	Promoter activated by dehydration, salt, cold and hormone treatments	Transcriptional upregulation	Orchestrates multi-stress response pathways; amplifies abiotic stress signaling cascades	CRISPRa	(Prabu and Theertha Prasad, 2012)
Drought	*SoP5CS*	Overexpression increases proline and drought tolerance	Gene knockout	Regulates proline biosynthetic pathways for osmotic adjustment under water deficit	CRISPR/Ca s9/Ca s12a; Base/Prime Editor	(Li et al., 2018)
Heat	*SsHsp17.9*	Induced under acute heat exposure	Transcriptional upregulation	Chaperone function; enhances cellular tolerance to acute heat stress	CRISPRa	(Tiroli and Ramos, 2007; Tiroli-Cepeda and Ramos, 2010)
Salinity	*ShPHT*	Salt-responsive transcript profile	Gene knockout	Regulates phosphate transport; maintains ion homeostasis in saline environments	CRISPR/Cas9/Cas12a; Base/Prime Editors	(Murugan et al., 2023)
Salinity, Low temperature	*SspNIP2*	Induced under salt and cold stress	Transcriptional upregulation	Aquaporin-mediated water transport enhances stress tolerance to salt and low temperatures	CRISPRa	(Park et al., 2015)
Salinity, Drought, Oxidative stress	*Scdr2*	Upregulated under salt, drought and oxidative challenge	Gene knockout/knock-in	Orchestrates cellular responses to drought, oxidative, and salt stressors	CRISPR/Cas9; Prime Editors	(Begcy et al., 2019)
Nutrient Deficiency (low-N)	*ScNRT2.3*	Upregulated under nitrogen limitation	Transcriptional upregulation	High-affinity nitrate transporter; enhances nitrogen uptake under low-nitrogen conditions	CRISPRa	(Hui et al., 2024)
(Category III)						
Heavy Metal (Cu)	*ScMT2-1-3*	Predicted function only	Gene knockout	Metallothionein; predicted copper detoxification	Base/Prime Editors	(Guo et al., 2013)

Validation status, Category I: Genes with direct CRISPR-editing experimental evidence in sugarcane. Category II: Genes functionally validated by non-CRISPR, molecular assays (overexpression, RNA-interference, transcript expression profiling) in sugarcane; CRISPR, approaches are only proposed. Category III: hypothetical candidate genes; support limited to sequence homology or cross-species data; no functional validation in sugarcane. Abbreviations: ABA, abscisic acid; ACLA, ATP-citrate lyase A; APX, ascorbate peroxidase; Cas9, CRISPR-associated protein 9; Cas12a, CRISPR-associated protein 12a; CRISPRa, CRISPR, activation; CRISPRi, CRISPR, interference; D27, DWARF27; Dir, dirigent protein; DREB, dehydration-responsive element-binding protein; GF, general regulatory factor (14-3-3 protein); GluD2, beta-1, 3-glucanase D2; H_2_O_2_, hydrogen peroxide; HSP, heat shock protein; JA, jasmonic acid; JAZ, jasmonate ZIM-domain; miRNAs, microRNAs; MT, metallothionein; MYB, myeloblastosis transcription factor; NAC, NAM/ATAF/CUC, transcription factor; NIP, nodulin 26-like intrinsic protein; NRT, nitrate transporter; nsLTP, non-specific lipid transfer protein; OE, overexpression; PEG, polyethylene glycol; P5CS, Δ^1^-pyrroline-5-carboxylate synthase; PHT, phosphate transporter; ROS, reactive oxygen species; RNAi, RNA, interference; SA, salicylic acid; Tpx2, thioredoxin peroxidase 2; WRKY, WRKY, transcription factor.

**TABLE 2:** Key target genes involved sugar beet responses to various abiotic stresses, genome editing approaches used to modulate these genes, their molecular and physiological functions under stress, and the optimized CRISPR/Cas tool suites applied for trait improvement.

Abiotic stress	Target gene	Observed phenotype	Genome editing approach	Molecular/Physiological function	CRISPR tool	References
(Category II)						
Drought	*BvDREB*	Transcript upregulated under drought and ABA treatments	Transcriptional up/downregulation	DREB-mediated drought signaling; regulates ABA-dependent stress response pathways	CRISPRa	(Qiu et al., 2024)
Heat	*BvHSF*	Heat-induced transcript accumulation	Transcriptional up/downregulation	Heat shock factor activity; induces HSP expression for cellular heat stress protection	CRISPRa/i	(Ismail et al., 2020)
Chilling/Cold	*BvRS1, BvRS2*	Cold-induced raffinose accumulation in taproots	Transcriptional up-/downregulation	Raffinose synthase activity; drives osmoprotection in cold-stressed cells	CRISPRa/i	(Kito et al., 2017)
Chilling/Cold	*GOLS2, GOLS3, BvRS2, BvRS5*	Elevated galactinol and raffinose under cold acclimation	Transcriptional up-/downregulation	Galactinol/raffinose synthesis; enhances cellular freezing tolerance	CRISPRa/i	(Keller et al., 2021)
Chilling/Cold	*BvSPL6, BvSPL3*	Cold-responsive expression in root and leaf tissues	Transcriptional up/regulation	SPL-mediated root development; augments cold stress tolerance in vegetative tissues	CRISPRa	(Xue et al., 2024)
Saline-Alkaline	*BvWRKY10, BvWRKY16*	Differential expressions under saline-alkaline stress	Transcriptional up-/downregulation	WRKY-mediated stress signaling; maintains ion homeostasis in saline-alkaline soils	CRISPRa/i	(Jiang et al., 2017)
Salinity	*BvbHLH93*	Salt-responsive transcript profile; reduced ROS when overexpressed	Transcriptional up-/downregulation	bHLH transcription factor activity; reduces ROS generation under salt stress	CRISPRa/i	(Wang et al., 2021b)
Salinity	*NAC21, NAC22, NAC100*	Salt-induced expression; post-transcriptional regulation via miR164	Gene knockout/knock in	NAC-mediated salt stress signaling; targeted by miR164 for post-transcriptional regulation	CRISPR/Cas9	(Cui et al., 2018)
Salinity	*BvNHX*	Salt-inducible expression in roots and leaves	Gene knockout/OE	Ion homeostasis; significantly upregulated by salt in roots and leaves	CRISPR/Cas9	(Wu et al., 2019b)
Salinity	*BvCIPK*	Upregulated under salt stress	Gene knockout/OE	Salt response; involved in signaling cascades for salt tolerance	CRISPR/Cas9	(Wu et al., 2023)
Drought, Salt, Heat	*BvNAC*	BvNAC40 expressed under drought; broad stress responsiveness	Gene knockout/OE	NAC transcription factors regulate growth, development, aging, and stress responses; *BvNAC40* is specifically expressed in response to drought stress	CRISPR/Cas9	(Sun et al., 2024)
(Category III)						
Drought + Chilling	*IWF5*	Predicted function only	Gene knockout/knock-in	Non-specific lipid transport enhances stress resistance to drought and chilling	Base/Prime Editors	(Kristensen et al., 2000)
Drought	*Bv_ralf, Bv_jkkr*	Predicted function only	Transcriptional upregulation	Initiates drought stress signaling cascades; modulates cellular stress acclimation	CRISPRa	(Stracke et al., 2014)
Chilling/Cold	*AtERDL6*	Predicted based on Arabidopsis homolog	Gene knockout	Early response to dehydration-like protein; predicted vacuolar carbohydrate trafficking	CRISPR/Cas9 knockout	(Yolcu et al., 2021; Reyer et al., 2024)
Alkaline	*EIN2*	Predicted function only	Gene knockout/knock-in	Ethylene signaling pathway modulation; enhances cellular tolerance to alkaline stress	CRISPR/Cas9 knockout	(Lei et al., 2011)
Heavy Metal (Ni)	*NIC3, NIC6, NIC8*	Predicted from wild beet homologs	Gene knockout	Cellular nickel detoxification; mediate tolerance to nickael contamination soils	Base/Prime Editors	(Bozdag et al., 2014)
Heavy Metal (Mn)	*BvHMA3, BmMTP10, BmMTP11*	Predicted from wild beet homologs	Gene knockout	Metal ion transport/chelation; drives manganese detoxification in vegetative tissues	Base/Prime Editors	(Erbasol et al., 2013)
Heavy Metal (Cd)	*BvNRAMP3*	Predicted based on homologs	Gene knockout	Metal ion transmembrane transport enhances cellular cadmium stress tolerance	Base/Prime Editors	(Haque et al., 2021)

Validation status, Category II: Genes functionally validated by non-CRISPR, molecular assays (transcript expression profiling, overexpression, RNA-interference) in sugar beet; CRISPR, approaches are only proposed. Category III: hypothetical candidate genes; support limited to sequence homology or cross-species data; no functional validation. Abbreviations: ABA, abscisic acid; bHLH, basic helix-loop-helix; CIPK, CBL-interacting protein kinase; CRISPRa, CRISPR, activation; CRISPRi, CRISPR, interference; DREB, dehydration-responsive element-binding protein; EIN2, ethylene-insensitive 2; ERDL6, early response to dehydration-like 6; GOLS, galactinol synthase; HMA, heavy metal ATPase; HSP, heat shock protein; HSF, heat shock factor; miR164, microRNA, 164; MTP, metal tolerance protein; NAC, NAM/ATAF/CUC, transcription factor; NHX, Na^+^/H^+^ exchanger; NIC, nicotianamine synthesis-related protein; NRAMP, natural resistance-associated macrophage protein; OE, overexpression; RALF, rapid alkalinization factor; ROS, reactive oxygen species; RS, raffinose synthase; SPL, SQUAMOSA, promoter-binding protein-like; WRKY, WRKY, transcription factor.

**TABLE 3:** Key target genes involved in sweet sorghum responses to abiotic stresses, detailed genome editing approaches used to modulate these genes, their molecular and physiological functions in stress acclimation, and optimized CRISPR/Cas tool suites applied for targeted trait improvement.

Abiotic stress	Target gene	Observed phenotype	Genome editing approach	Molecular/Physiological function	CRISPR tool	References
(Category I)						
Salinity + Drought	*SbBADH2*	CRISPR/Cas9 knockout produces fragrance (2-AP); enhances salinity and drought tolerance	Gene knockout/knock-in	Produces fragrance (2-AP); enhances salinity and drought tolerance	CRISPR/Cas9	(Cheng et al., 2024)
(Category II)						
Heat	*SbHsp70-1*	Heat-induced expression; protects cellular machinery from thermal stress	Transcriptional upregulation	HSP-mediated chaperone activity; protects cellular machinery from thermal stress	CRISPRa	(Mulaudzi-Masuku et al., 2015)
Chilling	*SbCBF6*	Increased expression during chilling stress; modulated by JA signalling	Transcriptional upregulation	CBF-mediated cold stress signaling; regulates JA biosynthetic pathways for chilling tolerance	CRISPRa	(Chopra et al., 2015)
Drought	*LTPL24*	Increased drought resistance in presence of PEG	Transcriptional upregulation	Lipid transfer protein activity; preserves membrane integrity under drought stress	CRISPRa	(Abdel-Ghany et al., 2020)
Drought	*SbLTP1.4, SbLTP1.5*	Distinct floral tissue-specific and stress-responsive promoters	Transcriptional upregulation	Non-specific lipid transfer proteins; preserve membrane integrity under drought stress	CRISPRa	Cossio et al., 2025
Saline-Alkaline	*SbWRKY50*	Negatively regulates salt stress responses; binds SOS1 and HKT1 promoters	Gene knockout/knock-in	WRKY-mediated ion homeostasis; negatively regulates cellular salt stress responses	CRISPR/Cas9; Base/Prime Editors	(Song et al., 2020)
Salinity	*NADP-ME*	Salt-induced expression; increases CO2 fixation and NADPH for ROS detoxification	Gene knockout/knock-in	Malic enzyme activity; boosts CO_2_ fixation and ROS metabolism in salt-stressed cells	CRISPR/Cas9/Cas12a; Base/Prime Editors	(Guo et al., 2018)
Salinity	*Sb04g030640*	Upregulated in leaves under salt stress; associated with ABA biosynthesis	Gene knockout/knock-in	ABA biosynthetic pathway modulation; initiates salt stress signaling cascades	CRISPR/Cas9/Cas12a; Base/Prime Editors	(Yang et al., 2017)
Salinity	*Sb09g006700 (PYL8)*	Downregulated in roots of salt-sensitive line; ABA signalling component	Gene knockout/knock-in	ABA signaling pathway component; mediates cellular salt stress responses	CRISPR/Cas9/Cas12a; Base/Prime Editors	(Yang et al., 2017)
Salinity	*Lhca1/2-4, Lhcb1-5/6*	Downregulation associated with enhanced salt stress tolerance	Gene knockout/knock-in	Light-harvesting complex activity; preserves photosynthetic efficiency under salt stress	CRISPR/Cas9/Cas12a; Base/Prime Editors	(Sui et al., 2015)
Salinity	*Sb02g002830, Sb09g021810*	Upregulated; crucial for stable assembly of oxygen-evolving complex and ATP synthase	Gene knockout/knock-in	Maintains oxygen-evolving complex/ATP synthase stability; preserves photosynthetic function in salt stress	CRISPR/Cas9/Cas12a; Base/Prime Editors	(Sui et al., 2015)
General abiotic stress	*NAC074A*	Non-functional allele linked to increased juice yield and total sucrose per plant	Transcriptional upregulation	NAC-mediated multi-stress response; enhances juice/sucrose yield	CRISPRa	(Xia et al., 2018)
(Category III)						
Heavy Metal (Al)	*SbWRKY22, SbWRKY65*	Induced in root apices under Al stress; expression validated in related species only	Gene knockout/knock-in	WRKY-mediated aluminum tolerance; reduces callose deposition in root apices	CRISPR/Cas9; Prime Editors	(Guan et al., 2023)
Heavy Metal (Cd)	*SbYS1, SbWRKY72*	Crucial for Cd tolerance and accumulation; expression validated in related species	Gene knockout/knock-in	Metal ion transport/transcriptional regulation; modulates cadmium tolerance/accumulation	CRISPR/Cas9; Prime Editors	(Jia et al., 2024)

Validation status, Category I: Genes with direct CRISPR-editing experimental evidence in sweet sorghum. Category II: Genes functionally validated by non-CRISPR, molecular assays (transcript expression profiling, overexpression, association mapping) in sweet sorghum; CRISPR, approaches are only proposed. Category III: hypothetical candidate genes; support limited to sequence homology or cross-species data; no functional validation in sweet sorghum. Abbreviations: 2-AP, 2-acetyl-1-pyrroline; ABA, abscisic acid; BADH2, betaine aldehyde dehydrogenase 2; bZIP, basic leucine zipper; CBF, C-repeat binding factor; CRISPRa, CRISPR, activation; CRISPRi, CRISPR, interference; EFM, early flowering MYB; GOLS, galactinol synthase; HSP, heat shock protein; Lhca, light-harvesting complex of photosystem I; lhcb, light-harvesting complex of photosystem II; LTP, lipid transfer protein; MYB, myeloblastosis transcription factor; NAC, NAM/ATAF/CUC, transcription factor; NADP-ME, NADP-dependent malic enzyme; OE, overexpression; PYL, pyrabactin resistance-like; ROS, reactive oxygen species; RS, raffinose synthase; SPL, SQUAMOSA, promoter-binding protein-like; WRKY, WRKY, transcription factor; YS1, yellow stripe 1.

### 3.2 Enhancing tolerance to drought, cold, and heat stresses

Sugar crops are significantly affected by various stresses that alter morphological, physiological, biochemical, and molecular characteristics (Pandey et al., 2025; Wang Z. et al., 2026). These stresses adversely affect the yield, growth, and development, causing physical damage and physiological disruption. Molecular non-specific lipid transfer proteins (nsLTPs) play a crucial role in facilitating lipid transport across cellular membranes as a plant-adaptive mechanism to drought stress (Table 2). In response to drought stress, plants upregulated the expression of *nsLTP* genes (Figures 3A,B). These proteins contribute to membrane integrity and stability by transferring lipids between membranes, thereby reducing water loss through transpiration and cellular dehydration (Liu et al., 2015; Giri and Kumar, 2026).

**FIGURE 3:**
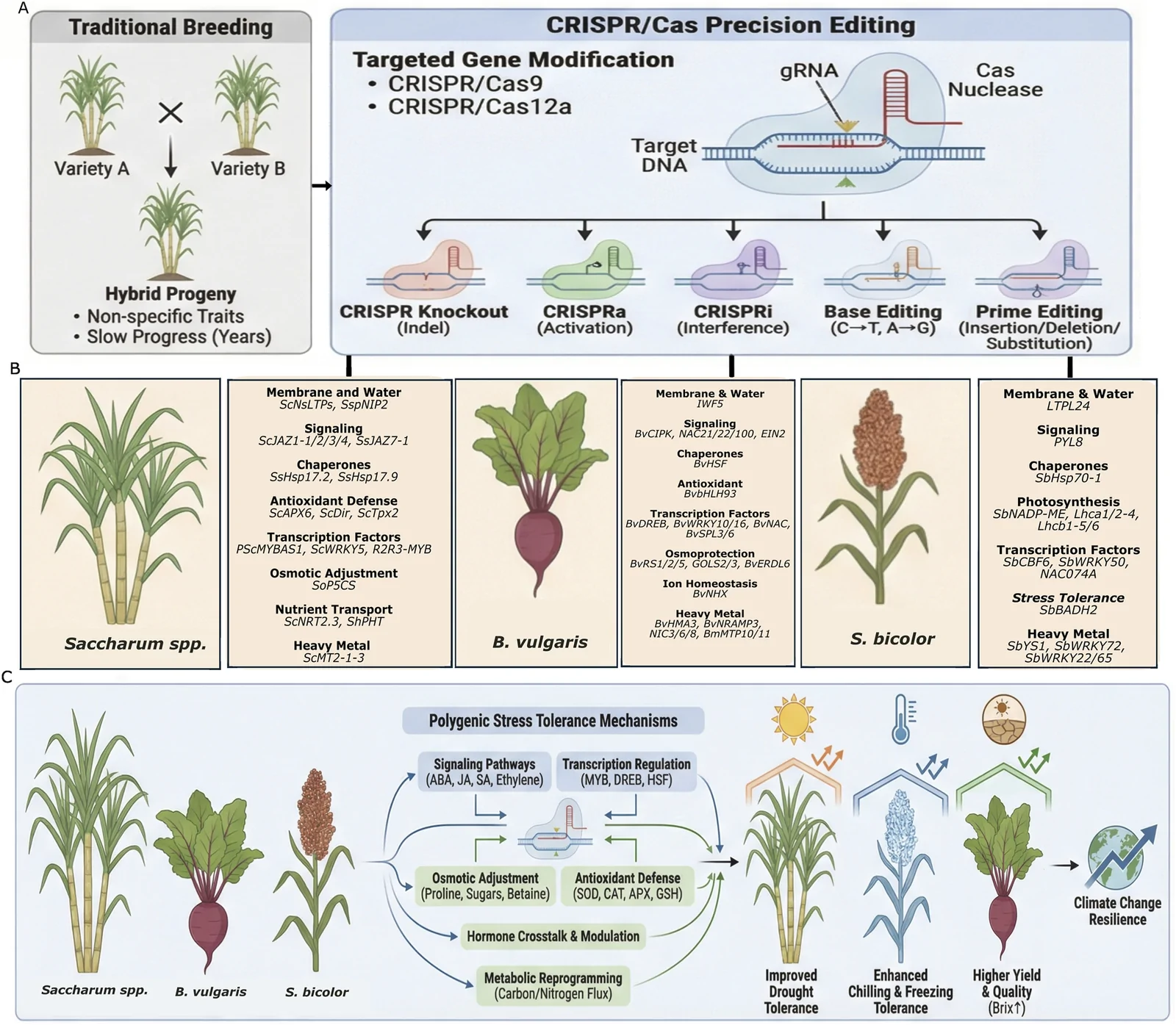
CRISPR/Cas precision editing strategies and candidate target genes for abiotic stress tolerance in sugar crops. **(A)** Traditional breeding versus CRISPR/Cas tools (Cas9, Cas12a) and editing outcomes (knockout, CRISPRa, CRISPRi, base editing, prime editing). **(B)** Candidate target genes organized by crop—sugarcane (*Saccharum spp.*), sugar beet (*B*. *vulgaris*), sweet sorghum (*S*. *bicolor*)—and functional category; *SbBADH2* is the only gene with direct CRISPR editing validation. **(C)** Polygenic tolerance mechanisms integrating stress signaling, transcription regulation, osmotic adjustment, antioxidant defense, hormone crosstalk, and metabolic reprogramming, leading to improved stress tolerance, yield, and climate resilience. SciDraw AI: Scientific illustration generator. Retrieved from https://scidraw.com/aidrawing; accessed on June 4, 2026.

Heat stress disrupts membrane and protein folding, leading to cell damage. *nsLTPs* have also been implicated in thermotolerance by stabilizing the membranes at high temperatures (Chen et al., 2017; Hussain et al., 2018). *S. spontaneum* produces soluble, small basic proteins (ScNsLTPs) involved in drought adaptive responses (Hussain et al., 2018). The *ScNsLTP* gene validated with upregulation under chilling and PEG stress, represents a high-priority target for CRISPRa-mediated overexpression to improve drought and cold resilience ((Chen et al., 2017; Li and Iqbal, 2024). The transcript of the *LTPL24* gene in *S. bicolor*, belongs to non-specific lipid transfer proteins, increases drought resistance in the presence of polyethylene glycol (PEG) (Abdel-Ghany et al., 2020). This suggests that genes encoding non-specific lipid transfer proteins can be targeted by the CRISPR/Cas system in sugar crops to develop drought tolerance (Table 3). Furthermore, the IWF5 protein, consisting of 92 amino acids, was isolated from sugar beet leaf intercellular fluid using cation-exchange chromatography and reverse-phase high-performance liquid chromatography. NsLTP has eight conserved cysteine residues in its amino acid sequence (Kristensen et al., 2000). The advent of CRISPR/Cas base editors presents a promising opportunity to enhance the utility of IWF5 in conferring valuable traits, such as drought and heat resistance, to sugar crops (Table 1).

Numerous TFs, including members of the v-myb avian myeloblastosis viral oncogene homologue (MYB) family and bZIP proteins, associated with resilience against stressors. Genes within the myeloblastosis (MYB) family are crucial for orchestrating plant responses to various abiotic stressors (Jiang et al., 2017). These TFs function as master regulators, modulating gene expression networks involved in stress perception, signal transduction, and subsequent physiological adaptations (Figures 3B,C). For example, *Sb08g019720*.*1,* associated with the SNP-8-50726311 locus, exhibits homology with the *Arabidopsis* EFM gene, a member of the MYB family of transcription factors. Similarly, *Sb01g037050*.1, linked to the SNP-1-60603003 locus, is homologous to a maize gene encoding a basic leucine zipper (bZIP). These findings suggest that the early flowering MYB protein (EFM) and bZIP genes play a significant role in the drought tolerance of sweet sorghum. These genes can be edited using CRISPR/Cas tools to enhance drought tolerance (Table 3). Similarly, the promoter of the *S. officinarum* stress-related MYB (*ScMYBAS1*) has been identified, which regulates abiotic stresses such as dehydration, salt, cold, and hormone treatments salicylic acid (SA) and methyl jasmonate) (Prabu and Theertha Prasad, 2012). Therefore, engineering the *PScMYBAS1* gene in sugar crops using CRISPRa can enhance tolerance to stress conditions.

In sugarcane, genes from *S. spontaneum* jasmonate zim domain 1/2/3/4 (*SsJAZ1-1/2/3/4*) were significantly upregulated under drought conditions. The transcript level of *ScJAZ1* was elevated in response to jasmonic acid (JA), polyethylene glycol (PEG), and abscisic acid (ABA). These genes are crucial candidates for sugarcane breeding, providing a foundation for enhancing drought stress tolerance (Table 2), through targeted gene modifications (Zhou et al., 2024). However, studies on *ScD27.2*’s role in drought-mediated tillering plasticity coordinates with the transcription factor *ScMYB44,* revealing a molecular module integrating strigolactone metabolism, plant architecture, and stress adaptation (Zan et al., 2023; Wu Z. et al., 2026). RNAi-mediated knockdown of *ScD27.2* increases tiller number and reduces drought tolerance, while overexpression suppresses tillering and enhances drought tolerance. A CRISPR/Cas9 knockout would phenocopy the RNAi knockdown line, resulting in increased tillering but compromised drought tolerance, which is the opposite of the desired outcome for enhancing drought resilience. Therefore, CRISPRa-mediated overexpression of *ScD27.2* represents the appropriate strategy to enhance drought tolerance while reducing tillering. Alternatively, promoter editing or base editing could be employed to achieve more subtle modulation of *ScD27.2* expression, potentially uncoupling the trade-off between tillering and stress tolerance. Similarly, *B. vulgaris BvMYB* genes, such as *Bv Rapid alkalization* factor (*ralf*) and *Bv_jkkr,* have shown targets for CRISPR/Cas editing, indicating a pathway for precise genetic modifications in sugar beet to enhance drought resistance (Stracke et al., 2014).

Genes associated with heat shock factors (*HSF*s) responded to heat stress (Figures 3B,C). HSFs activate the transcription of heat shock genes, including heat shock proteins (HSPs), which are essential for maintaining cellular homeostasis and protecting plants against stress-induced damage. In sugarcane, heat stress induces HSF genes, particularly members of the HSF-A and HSF-B classes, whereas BvHSF genes in sugar beet are responsive to both heat and salt stresses. These stress-responsive HSFs therefore represent potential targets for genetic manipulation to enhance abiotic stress resilience in sugar crops. (Ismail et al., 2020; Zhang J.-L. et al., 2024). The CRISPRa system employs dCas9 fused to transcriptional activator domains (e.g., *VP64*) to upregulate *BvHSF* expression without inducing DNA breaks, potentially enhancing heat resistance. Moreover, transcriptomic analyses revealed upregulation of *HSF* genes in sweet sorghum under heat stress. Using the CRISPR/Cas system, targeted editing of the *SbHsp70-1* gene in sweet sorghum can improve heat resistance (Mulaudzi-Masuku et al., 2015; Misra et al., 2023). The heat- and drought-inducible- activities of heterologous sHSP promoters, particularly stems, correlate with the native expression and water stress-response of endogenous class I small *HSP* genes, supporting the conserved role of HSP-mediated-regulatory networks in sugarcane stress adaptation. Similarly, *SsHsp17.2* and *SsHsp17.9*, classified as class I *Hsps*, expressed *in vitro* in sugarcane. Furthermore, the *SsHsp17.2* gene is induced in sugarcane under water stress (Tiroli and Ramos, 2007; Tiroli-Cepeda and Ramos, 2010; Qiande and Altpeter, 2025). Genes encoding small heat shock proteins, including *SsHsp17.2* and *SsHsp17.9*, have been validated at the transcriptional level in sugarcane (Tiroli and Ramos, 2007; Tiroli-Cepeda and Ramos, 2010; Qiande and Altpeter, 2025). CRISPRa-mediated activation of these genes represents a promising strategy to enhance heat-stress resilience. Furthermore, the DREB-family plays a pivotal role in conferring drought tolerance (Figure 3) (Zhang and Xia, 2023). In a recent investigation, 30 *BvDREB* family genes were classified into six distinct subfamilies in sugar beets. *The BvDREB* genes were responsive to drought stress and exhibited varying expression patterns. These findings contribute to drought tolerance by regulating ABA signaling pathways, proline biosynthesis, and hormone levels (Qiu et al., 2024). Similarly, 11-DREB1/CBF genes in cold-tolerant *Erianthus fulvus* confirmed cold-responsive genes, and validated that *EfDREB1C* acts as a nuclear transcriptional activator enhancing cold tolerance (Qian et al., 2025), by regulating multiple stress-related pathways, providing gene targets for sugarcane cold resistance. Collectively, these findings represent promising targets for gene editing using the CRISPR/Cas system to enhance the abiotic tolerance of sugar crops.

Worldwide, crops are subjected to severe cold spells and simultaneously face drought conditions or impaired water transport and absorption during the cold season. The simultaneous occurrence of chilling and drought stress substantially hinders plant development and poses a significant threat to crop yields (Chan et al., 2025; Yanqing et al., 2026). These stressors severely affect the yield, growth, and development of sugar crops, leading to morphological, physiological, and biochemical changes, as well as molecular modifications (Figures 2, 3). Taken together, *nsLTPs* are indispensable components of plant defense mechanisms against adverse stress. Transcript analysis revealed the upregulation of the *ScNsLTP* gene under chilling and PEG stress in sugarcane, indicating a stress response to low temperatures (Chen et al., 2017). Genome-wide investigations have identified 71 *nsLTP* genes in *Saccharum* spp. that exhibit distinct expression patterns in response to salicylic acid treatment (Li J. et al., 2022), providing gene targets for improving stress tolerance in sugarcane. The *SsGF2.1–2.4* family genes were identified to be expressed in various tissues in response to cold stress (Zhang H.-L. et al., 2024). These genes belong to the 14-3-3 protein family, a group of highly conserved acidic proteins in sugarcane. In *S. bicolor*, two *NsLTP* genes, *SbLTP1.4* and *SbLTP1.5*, exhibit distinct floral tissue–specific and stress-responsive promoters (Cossio et al., 2025), suitable for driving efficient CRISPR-mediated genome editing.

The reduced expression of BvLTP-1 is associated with mitochondrial dysfunction under cold stress in sugar beet (Matsuhira et al., 2007). CRISPRa could be employed to restore or enhance BvLTP-1 expression, thereby preserving mitochondrial function and improving chilling tolerance. Alternatively, may dominant-negative isoform is responsible, base editing could correct the regulatory variant. In sugar beet, increased raffinose accumulation in the root pith contributes to freezing tolerance, highlighting the protective role of raffinose under cold stress. Raffinose-family oligosaccharides (RFOs) are also important for cold acclimation, with galactinol synthase (GOLS) acting as a key regulator of their biosynthesis and representing a potential target for genetic manipulation.(Keller et al., 2021). The isolation and characterization of *BvRS1* and *BvRS2*, genes encoding raffinose synthase (RS), a key enzyme showed that chilling stress induces transcription in leaves and roots (Kito et al., 2017). High expression of *GOLS2, GOLS3, BvRS2*, and *BvRS5* has been observed under cold conditions (Keller et al., 2021). Notably, the *BvRS5* gene and raffinose content showed significant increases during freezing in taproots of tolerant sugar beet varieties, indicating enhanced expression of *GOLS* and *RS* genes, for freezing resistance (Keller et al., 2021; Yolcu et al., 2021; Pandey et al., 2025). Consequently, targeting *GOLS* and *RS* genes using CRISPRa (dCas9-transcriptional activator fusion) may enhance the resistance of sugar beet varieties to freezing stress by upregulating raffinose biosynthesis without introducing permanent genomic alterations. Notably, membrane proteins are crucial for detecting cold stress and initiating cytosolic Ca^+2^ signals, which may regulate downstream transcription factors.

Squamous promoter-binding protein (*SPL*) transcription factors play a crucial role in regulating plant growth and development. *BvSPL6* expresses mature roots under abiotic stress. Similarly, *BvSPL3* in sugar beets enhances resistance to cold. *BvSPL3* contributes to root growth and sugar accumulation. The CRISPR-Cas system allows the targeting of these genes to improve cold stress in sugar beets. By editing the *BvSPL6* and *BvSPL3* genes using CRISPRa, their expression can be enhanced, thereby increasing plant resilience to cold stress. For example, overexpression of *BvSPL6* could enhance root development and cold stress, and modification of *BvSPL3* could optimize root growth and sugar accumulation during plant maturation. Targeted genetic interventions using the CRISPR/Cas system can significantly enhance the overall cold tolerance and productivity of sugar beets through the inherent functions of *SPL* genes (Xue et al., 2024). In sweet sorghum, *SbCBF6* exhibited increased expression levels during chilling stress. The regulation of CBF expression is modulated by JA signaling. Notably, in chilling-tolerant sorghum, key genes involved in JA biosynthesis, such as allene oxidase synthase and 12-oxophytodienoic acid reductase, were upregulated. This finding indicates that JA biosynthesis plays a crucial role in imparting chilling tolerance (Chopra et al., 2015; Marla et al., 2017; Namata et al., 2025). Additionally, a massively parallel reporter assay was employed to systematically evaluate 30,000 CRISPR-accessible mutations, including deletions, substitutions, and motif insertions, across the native promoters and 5′untranslated regions of three photosynthesis genes; *PsbS*, *Raf1*, and *SBPase*. These results showed that gene expression is modulated within a ∼500-bp core promoter region, with mutational effects that are reproducible and protein accumulation. Notably, compact deletions and targeted motif insertions in these regions enhanced protein production 30-fold- relative to the wild type (Groover et al., 2026). Since mutation-effect-relationships were gene-specific. These findings highlight the need for customized regulatory maps. Thus, a high-throughput-cis-regulatory fine-mapping-approach enables targeted, minimal, and non-transgenic CRISPR edits to advance the improvement of sugar crops.

### 3.3 Improving saline and alkaline stress tolerance

Salinity presents significant challenges to sugar crops, leading to hyperosmotic and ionic imbalances that affect crop production, particularly in tropical and subtropical regions (Figure 4). *ScGluD2*, a novel member of the subfamily D beta-1,3-glucanase, acts as a stress-responsive gene in sugarcane and is expressed as a defense mechanism against smut fungi and saline stress (Wu et al., 2022). The expression of *ScGluD2* transcripts increased after 12 h of salt stress. The transcripts were upregulated between 6 and 24 h following ABA, H_2_O_2_, and CdCl_2_ treatment, indicating that ABA may function as a signaling molecule regulating oxidative stress and play a pivotal role under salt stress (Su et al., 2016). ScGluD2 transcript upregulated under salt stress can be targeted for CRISPRa-mediated overexpression to confer saline stress resistance in sugarcane. Various TF family genes, such as WRKY, NAC, and MYB have been identified to contribute to abiotic stress tolerance (Zhang et al., 2022). Under saline-alkaline conditions, WRKY family genes can be activated through CRISPRa activation or downregulated via CRISPRi interference, targeting genes involved in stress responses and mitigating the adverse effects of stress on plants. Additionally, WRKY (TFs) enhance the antioxidant defense system of plants by regulating genes encoding antioxidant enzymes, such as superoxide dismutase (SOD), catalase (CAT), and peroxidases (POD) (Zhang et al., 2022; Zeng et al., 2024; Yang L. et al., 2025). The detoxification of reactive oxygen species (ROS) protects plant cells from oxidative damage (Figures 3C, 4). These genes can also regulate the expression of osmolyte biosynthesis genes, leading to the accumulation of proline, glycine, betaine, and sugars (Zeng et al., 2024). These compounds contribute to osmotic adjustment and cellular protection, while coordinated regulation of ion transport systems maintains ion homeostasis by promoting the retention and compartmentalization of essential ions such as K^+^ and the exclusion or sequestration of toxic ions.

**FIGURE 4:**
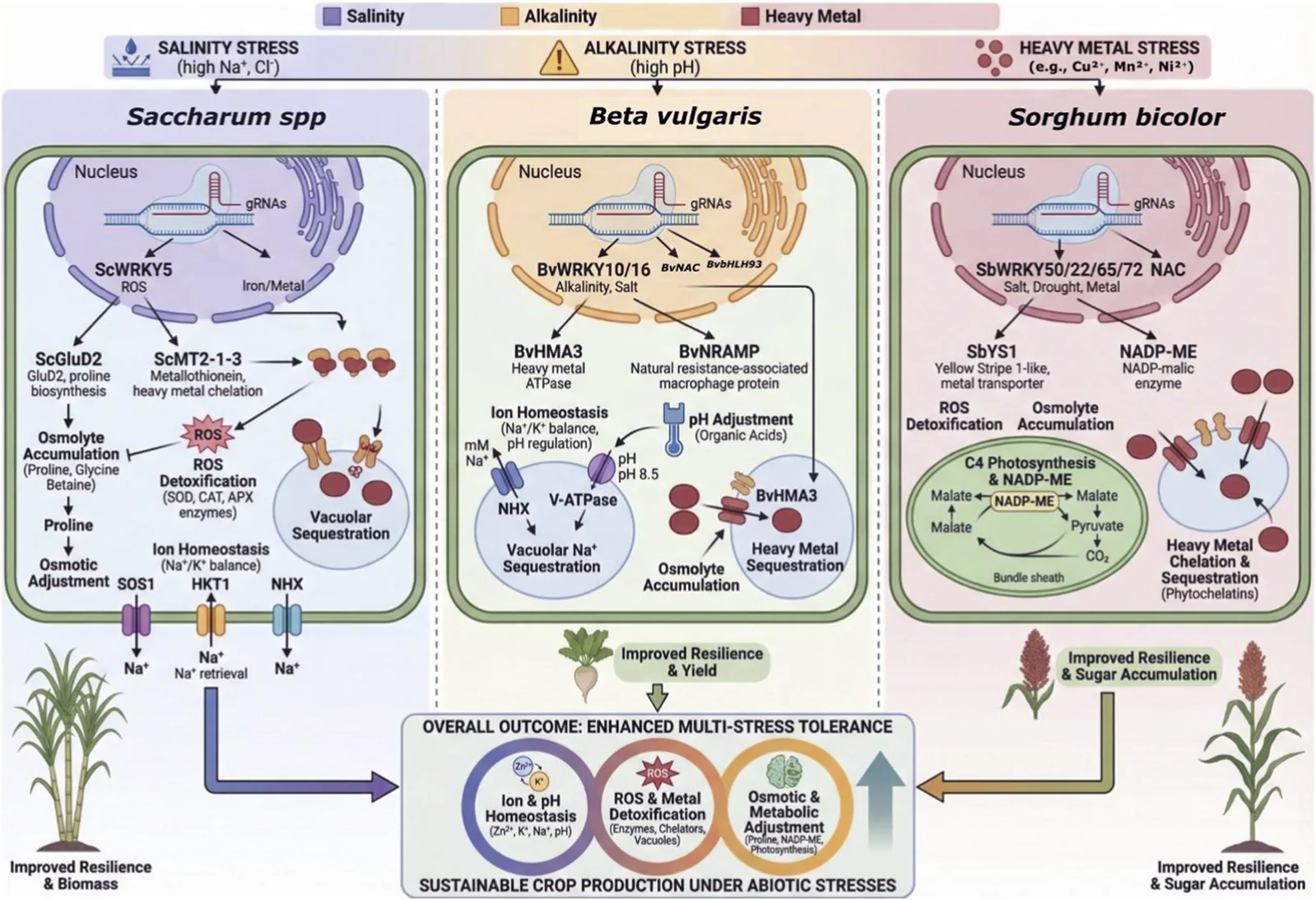
Schematic overview of CRISPR-Cas9 strategies to improve salinity, alkalinity, and heavy metal stress tolerance in sugar crops. Targeted transcription factors (*WRKY, NAC,* and *bHLH*) and functional genes that modulate core stress-adaptive pathways, including ion homeostasis, pH regulation, reactive oxygen species (ROS) detoxification, heavy metal chelation/sequestration, osmolyte accumulation, and C4 photosynthesis. Crop-specific targets: sugarcane (*ScGluD2*, *ScWRKY5*); sugar beet (*BvWRKY10/16*, *BvbHLH93*); sweet sorghum (*SbWRKY50*, *NADP-ME*, *SbBADH2*). These edits enhance multi-stress tolerance, boost biomass and sugar yield, and support sustainable sugar crop production, thereby demonstrating the translational potential of CRISPR in sugar crop improvement. SciDraw AI: Scientific illustration generator. Retrieved from https://scidraw.com/aidrawing; accessed on June 24, 2026.

Elevated pH levels in alkaline soils disrupt cellular pH stability, cell membrane integrity, and reduce root vigor and photosynthetic activity in sugar beets. Under saline stress, cultivated sugar beet varieties exhibit increased antioxidant enzyme activity (Wang et al., 2017; Li et al., 2020; Rao et al., 2023). Homeostasis of K^+^ and Na^+^ is a critical adaptation strategy for sugar beets under alkaline stress (Houmani and Corpas, 2026). The identification of 58 *WRKY* genes, nine of which respond to alkaline stress in sugar beet shoots and roots. Elevated expression of *BvWRKY10* (in terminal and lateral shoots) and *BvWRKY16* (in roots) under alkaline stress. A significant increase in Na^+^ concentration in the shoots and roots of sugar beets under alkaline conditions, increasing sodium bicarbonate concentrations; keeping, potassium ion concentrations stable (Wu et al., 2019a). Manipulation of *BvWRKY* gene expression using CRISPR/Cas tools may yield stress-resistant sugar beet varieties (Table 1). These genes play pivotal roles in sugar beet development and alkaline stress.

Long noncoding RNAs (lncRNAs) and *bHLH* genes in sugar beets respond to alkaline and salt stress, respectively. For example, *BvbHLH93* mitigates reactive oxygen species (ROS) generation and regulates polyamine metabolism during salt stress (Wang Y. et al., 2021). Similarly, the expression of *Sporisorium scitamineum ScWRKY5* is significantly altered in response to various stressors, including inoculation, salicylic acid, abscisic acid, polyethylene glycol, and sodium chloride (Wang et al., 2020). Notably, after inoculation with *S. scitamineum*, resistant sugarcane varieties exhibited increased *ScWRKY5* expression, whereas susceptible varieties showed decreased expression. CRISPRa technology can be used to edit *ScWRKY5* by targeting specific regions to enhance its activity under saline and alkaline stress. The responsiveness of plants to these stresses can be augmented by introducing mutations or modifications through base or prime editing, thereby improving the resilience and productivity of sugarcane under saline and alkaline conditions.

*SbWRKY50* from sweet sorghum negatively influences salt stress responses in *A. thaliana* by regulating ion homeostasis by binding to the upstream promoter of *SOS1* and *HKT1* genes, thereby offering valuable insights into enhancing saline stress tolerance in sweet sorghum using the CRISPR/Cas system (Song et al., 2020). Conversely, NAC may modulate the expression of genes involved in osmotic adjustment, ion uptake and compartmentalization, maintenance of cell integrity, and stress signaling pathways. In sweet sorghum, the non-functional gene, *NAC074A,* has been linked to increased juice yield, thereby increasing the overall sugar content. However, genetic variation did not alter the sucrose concentration or Brix of the juice. Instead, resulted in a higher total juice volume per plant (Xia et al., 2018). This led to a greater total amount of sucrose per plant, enhancing the value of the plant for sugar production. This gene can be targeted using CRISPRa to increase the overall sugar productivity of sweet sorghum under abiotic stress (Table 3).

In sugar beet salt-tolerant cultivars, the expression of *NAC21*, *NAC22*, and *NAC100*, were targets of miR164, exclusively induced under salt stress (Cui et al., 2018). Moreover, NAC family genes involved in stress response regulation using CRISPR/Cas technology could be highly beneficial for enhancing tolerance to salt and alkaline stress in sweet sorghum, sugarcane, and sugar beet plants. Through precise gene editing, researchers can elucidate the specific roles and regulatory mechanisms of NAC (TFs) in plant stress adaptation, thereby improving the yield, stability, and productivity. MYB (TFs), may regulate the expression of genes involved in osmolyte synthesis, stomatal closure, hormonal signaling, and metabolic adjustments to enhance stress tolerance (Wu et al., 2024). Gene expression patterns related to salt resistance in sweet sorghum identified *MYB*-type TFs as specific to salt-tolerant sorghum varieties. These *MYB*-type TFs are crucial for the synthesis of secondary metabolites and environmental stress (Chen et al., 2022; Zhou et al., 2025). The diverse expression profiles of *MYB*-type TFs may contribute to variations in gene response patterns associated with secondary metabolite synthesis in salt-tolerant sorghum genotypes. Investigating the regulatory mechanisms of these genes using CRISPRa or CRISPRi systems could enhance the salt and alkaline stress tolerance of sugar beets and sweet sorghum. Similarly, salt stress upregulates the gene encoding NADP-dependent- malic enzyme (NADP-ME) in sweet sorghum (Guo et al., 2018). This increases CO_2_ and pyruvate levels, boosting CO_2_ fixation efficiency, whereas it produced NADPH supports ROS detoxification (Mittler et al., 2022). Targeted editing of NADP-ME genes using CRISPR/Cas technologies offers a promising strategy to improve photosynthetic performance, salt tolerance and productivity in sugar crops under saline conditions.

The gene *Sb04g030640*, associated with ABA synthesis in sweet sorghum, was upregulated in the leaves. In addition, genes involved in the ABA signaling pathway, such as pyrabactin resistance (PYR)/pyrabactin resistance-like (PYL) and type 2C protein phosphatase (PP2C), exhibited distinct expression patterns. Specifically, the *PYL8*-encoding gene *Sb09g006700* was downregulated in the roots of the salt-sensitive line, whereas PP2C-encoding, gene were upregulated in the salt-sensitive line but downregulated in the salt-tolerant line of sweet sorghum (Yang et al., 2017). CRISPR/Cas tools can be used to fine-tune the expression of genes involved in the synthesis and signaling of jasmonic acid (JA), indole-3-acetic acid (IAA), and cytokinins (CK), thereby ensuring optimal hormonal balance and stress response. These genetic modifications can collectively improve the resilience of sweet sorghum to saline environments and enhance its growth and productivity. In contrast, the downregulation of genes encoding light-harvesting proteins, specifically *Lhca1* and *Lhcb1-5*, is associated with enhanced salt stress tolerance in sweet sorghum plants. Moreover, genes encoding *Lhca2-4* and *Lhcb6* are integral for controlling tolerance to saline conditions. Additionally, the upregulation of *Sb02g002830* and *Sb09g021810* is crucial for the stable assembly of the oxygen-evolving complex and ATP synthase, respectively, and contributes to the stability of the photosynthetic system during salt stress (Sui et al., 2015; Yang et al., 2020). Taken together, these genes are potential targets for CRISPRa tools to enhance salt stress tolerance in sweet sorghum.

### 3.4 Heavy metal stress tolerance

The exposure of plants to toxic heavy metals induces a range of metabolic and physiological changes that are contingent upon the concentration of these metals in the soil and specific plant species and varieties (Sperdouli, 2022; Ghuge et al., 2023). The development of sugar crops with enhanced tolerance to heavy metal stress is essential to sustain productivity and ensure the safety of agricultural products (Figure 4). The CRISPR-Cas system provides robust tools for achieving this objective through precise genome editing. A metallothionein gene (*ScMT2-1-3)*, consisting of 700 and 240 bp open reading frames (ORF) that encode 79 amino acid residues (Guo et al., 2013), plays a critical role in copper detoxification. In wild sugar beets, genes such as *BmMTP10*, *BmMTP11*, *NIC3*, *NIC6*, *NIC8*, *BvHMA3*, and *BvNRAMP3* are implicated in heavy metal stress tolerance (Erbasol et al., 2013; Bozdag et al., 2014; Haque et al., 2021), indicating potential target genes for CRISPR bases or prime editing to develop heavy metal-resistant sugar beet varieties.

In sweet sorghum, *SbWRKY22* and *SbWRKY65* transcripts were induced in the root apices under aluminum (Al) stress. These WRKY proteins are localized in the nucleus and exhibit transcriptional activity. Notably, *SbWRKY22* played a significant role in regulating key genes associated with Al tolerance, whereas *SbWRKY65* primarily affected the expression of *SbWRKY22*. This suggests that *SbWRKY65* may indirectly regulate Al tolerance genes through *SbWRKY22*. These findings indicate the presence of pathways mediated by *SbWRKY22* and *SbWRKY65* that regulate Al tolerance in sweet sorghum (Guan et al., 2023). Furthermore, *SbYS1* and the upstream transcription factor *SbWRKY72* are crucial for cadmium (Cd) tolerance and accumulation in sweet sorghum (Jia et al., 2024). Taken together, genes including *SbYS1* and *SbWRKY72* have been identified through transcriptomic analysis in sweet sorghum under Cd stress and show homology to known metal tolerance genes. These represent hypothetical targets requiring experimental validation before CRISPR-mediated editing. Moreover, transcriptomic analysis revealed that Arg treatment significantly induced genes involved in GSH biosynthesis, polyamine metabolism and cell wall remodeling, including pathways related to pectin synthesis and lignin polymerization, thereby promoting Cd sequestration and strengthening the cell wall. (Ye et al., 2025; Liu et al., 2026). Furthermore, investigating the regulatory mechanisms of these genes using CRISPR/Cas9/12 and prime-editing tools will enhance heavy metal stress tolerance in sugarcane, sugar beets, and sweet sorghum (Tables 1–3).

## 4 Challenges and translational barriers

Despite recent progress in genomics that sufficiently supports gene editing breeding (Supplementary Table S1), the actual deployment of CRISPR/Cas in sugar crops—predominantly sugarcane—faces a dominant translational bottleneck rooted in extremely low genetic transformation efficiency (Xia et al., 2018). Elite commercial sugarcane varieties display profound recalcitrance toward both *A. tumefaciens* mediated transformation and biolistic particle bombardment, with stable editing recovery rates of only 1%–5% across commercial varieties (Xia et al., 2018; Antony Ceasar and Ignacimuthu, 2023).This transformation barrier is multi-layered and genotype-dependent, driven by distinct physiological and epigenetic characteristics inherent to polyploid crops. Mature stalk and leaf explants show a limited capacity for embryogenic callus induction; ploidy-induced epigenetic silencing suppresses the uptake of exogenous DNA or RNP in proliferating cells; and tissue-specific cell wall rigidity hinders vector penetration during transfection. (Urnov et al., 2010). Combining low delivery and somatic regeneration pipeline of 6–12 months per editing event, a timeline far exceeding diploid model crops such as *Arabidopsis* and rice. Such prolonged culture cycles drastically inflate labor costs, slow germplasm screening, and delay multi-location field trail validation of stress-resistant edited lines. Even with haplotype-resolved gRNA design by sugarcane pangenome resources, the probability of isolating a uniform, heritable edited regenerant remains negligible without improvements to delivery workflows. Conceptually, advances in sugar crop pan-genomics and target gene identification have substantially advanced ‘which genes to modify’ challenge by enabling haplotype-aware guide design and the identification of stress-responsive gene families. However, the complexity of sugarcane’s auto-allopolyploid genome (∼10 Gb, 100–130 chromosomes), gene discovery remains an ongoing process, with many stress-responsive genes and regulatory networks yet to be fully characterized. Nevertheless, inefficient transformation and slow tissue culture regeneration create a persistent ‘how to deliver edits’ translation gap that remains the primary bottleneck for commercial deployment. Without transformative innovations in DNA-free ribonucleoprotein (RNP) delivery, lipid nanoparticle carriers, or plant viral vector transfection platforms, the full potential of CRISPR-engineered multi-stress resilient sugar varieties is challenging to be translated from lab experimentation to commercial breeding program. The application of CRISPR/Cas tools has attracted considerable interest to develop more resilient varieties. However, additional challenges beyond delivery also impede precise applications (Figure 5). The Cas9: gRNA complex can induce DNA breaks at off-target sites, potentially resulting in unintended mutations (Mall et al., 2024). The polyploid nature of sugar crops complicates mutant screening because numerous mutants are required for functional research. Moreover, high haplotype diversity means that multiple haplotypes exist for key agronomic genes (e.g., sucrose synthase), and editing a single copy often fails to produce a significant phenotype (Liu et al., 2023).

**FIGURE 5:**
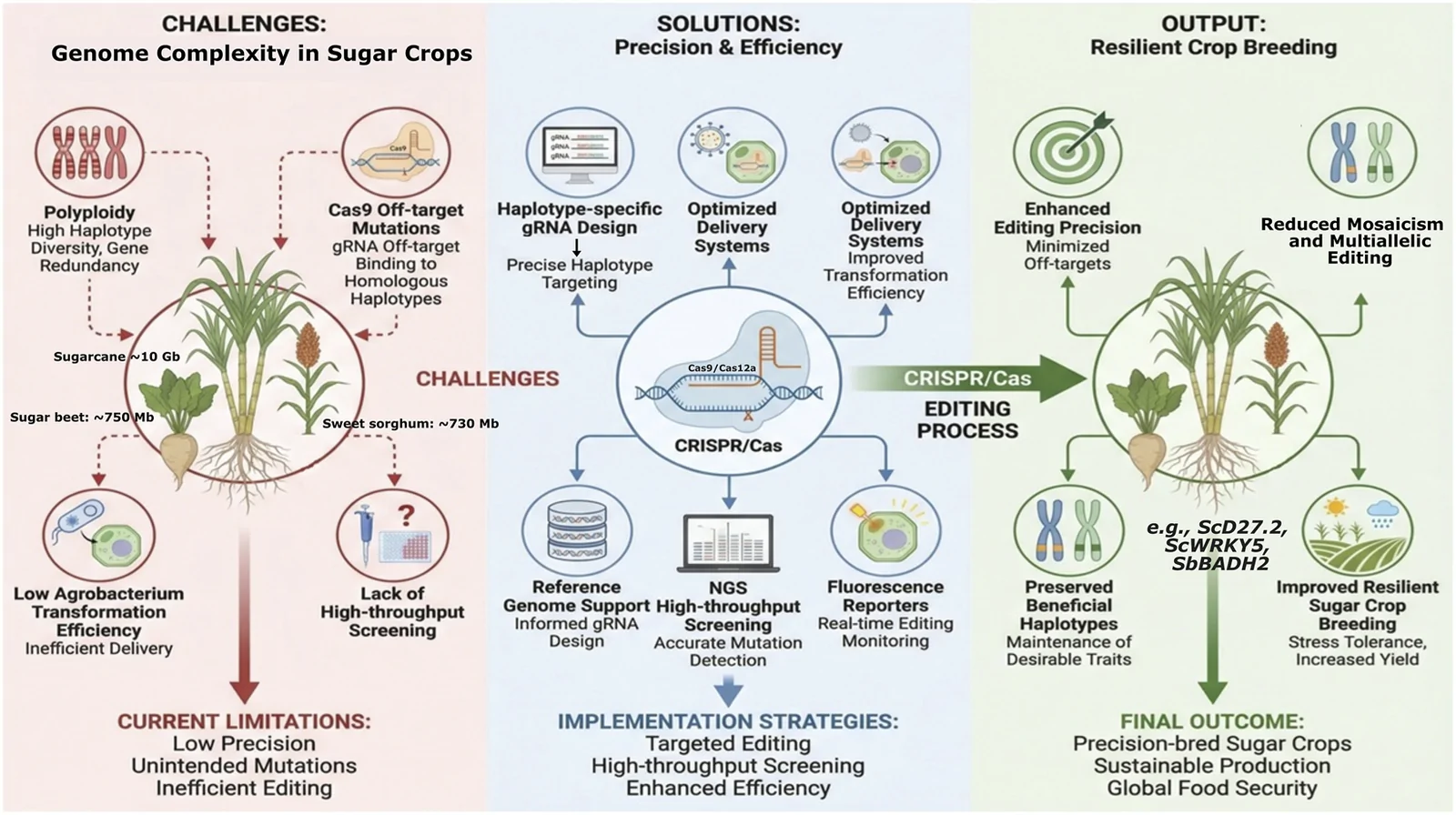
Overview of CRISPR/Cas genome editing in sugar crops. Illustrating the core challenges (polyploidy, haplotype diversity, off-target mutations, low transformation efficiency, precision solutions (haplotype-specific gRNA design, optimized delivery, high-throughput screening), and outcomes (enhanced editing precision, preserved beneficial haplotypes, resilient crop breeding for sustainable production, and global food security). Specific to polyploid sugar crops (Supplementary Table S1): sugarcane (∼10 Gb, 100–130 chromosomes, auto-allopolyploid); sugar beet (amphidiploid); sweet sorghum (diploid reference genome available) SciDraw AI: Scientific illustration generator. Retrieved from https://scidraw.com/aidrawing; accessed on 4 June 2026.

CRISPR off-target occurrences arise due to partial base pairing between gRNA and non-target genomic protospacers, with Cas9 allowing up to 1–4 mismatches in the crucial PAM-proximal seed region of the RNA-DNA hybrid duplex (Sternberg and Doudna, 2015). This risk is significantly heightened in polyploid sugar crops, particularly sugarcane, 10 Gb auto-allopolyploid genome, numerous segmental duplications, repetitive intergenic sequences, and hundreds of paralogous stress gene haplotypes (Ou, 2026; Wang J. et al., 2026). The gRNAs that bind conserved regions shared across homologous sub-genomes frequently cause unintended double-strand breaks and indel mutations, with three major consequences for variety development. First, off-target disruption of key sucrose-metabolism or stress-related genes (SPS, WRKY, DREB) can both weaken the intended stress-tolerance phenotype and lower sucrose accumulation—directly undermining the crop’s primary economic value. yield in stalks and taproot tissues, thereby negating the primary economic trait of sugar crops. Second, unintended mutation of metal sequestration genes, such as *ScMT2-1-3*, results in increased accumulation of toxic heavy metals (Cu, Cd) in edible sink tissues, thereby breaching global safety standards for food and feed, particularly in industrial sucrose and bioethanol feedstock (Guo et al., 2013). Third, global regulatory frameworks for gene editing categorize heritable off-target variants as uncharacterized genomic alterations, necessitating expensive multi-generational whole-genome sequencing (WGS) validation to achieve variety registration (Liberty et al., 2025). In sugarcane, regeneration cycle for each edited event, comprehensive off-target screening significantly extends breeding timelines and elevates germplasm development costs. In polyploids, mosaic chimerism resulting from uneven off-target cleavage across divergent haplotypes further leads to inconsistent stress resistance phenotypes (Eid et al., 2021).

### 4.1 Transformation and regeneration bottlenecks

Polyploidy imposes two inherent molecular constraints that exacerbate the delivery bottleneck, which is absent in diploid model crops. First, sugarcane embryogenic callus cells exhibit ploidy-dependent epigenetic silencing pathways, which inhibit the stable nuclear uptake and retention of either plasmid CRISPR constructs or Cas9-gRNA ribonucleoproteins (RNPs) (Birch et al., 2010). This epigenetic suppression significantly reduces the proportion of callus that sustain functional editing machinery. Second, severe gene dosage imbalance across divergent haplotypes disrupts cellular reprogramming during somatic embryogenesis. Edited polyploid cells exhibit irregular mitotic division, prolonging full plant regeneration by an additional 3–6 months compared to diploid crops (Li and Iqbal, 2024; Mall et al., 2024). The combination of extremely low transformation recovery and extended tissue culture timelines impedes high-throughput screening of extensive stress-tolerant germplasm screening (Antony Ceasar and Ignacimuthu, 2023), even when gRNA sequences are fully optimized using haplotype-resolved pangenome assemblies.

### 4.2 Functional redundancy and haplotype diversity

Significant sequence divergence among paralogous gene haplotypes constitutes a translational barrier that is exclusive to polyploid sugar crops. In sugarcane, 71 distinct *nsLTP* haplotypes collectively regulate membrane stability under drought and chilling conditions (Li J. et al., 2022). Yet targeted editing of just one or two haplotypes rarely produces a detectable phenotype in transpiration control or cold acclimation, since the unedited paralogs continue to compensate functionally. Similar multi-copy redundancy is evident in sugar beet raffinose synthase (*BvRS*) and sweet sorghum salt-responsive *WRKY* genes. This genetic redundancy poses two primary challenges for breeding. First, custom multiplex gRNA cocktails must be developed to align with the unique single nucleotide polymorphism of each functional haplotype copy of a target gene (Kumbara et al., 2025). Second, high-depth next-generation sequencing genotyping is essential to verify uniform editing across at least three to five paralogous copies per regenerated plant, significantly increasing sequencing costs and screening efforts for breeding.

### 4.3 DNA-Free RNP delivery

CRISPR editing relies on plasmid vectors encoding Cas9 and gRNA sequences, which are delivered into plant cells and transcribed endogenously to assemble the editing complexes. In contrast, DNA-free ribonucleoprotein (RNP) delivery directly introduces pre-assembled Cas9–guide RNA ribonucleoprotein complexes into sugar crop cells through biolistic bombardment, PEG-mediated protoplast transfection, or other membrane-permeabilization methods (Urnov et al., 2010; Zhang et al., 2020). This technology is designed to fully eliminate exogenous DNA templates or expression plasmids, thereby preventing the integration of foreign genetic material into the sugar crop nuclear genomes. After cellular uptake, nuclear localization signals (NLS) fused to recombinant Cas9 guide the RNP complex into the nucleus, where gRNA hybridizes to the target protospacers and Cas9 catalyzes site-specific double-strand breaks (DSBs). A defining functional feature of RNPs is transient intracellular activity: unbound Cas9 protein and free gRNA are rapidly degraded by endogenous plant proteases and nucleases within 2–5 days post-transfection, with no sustained production of editing machinery across cell divisions (Eid et al., 2021; Kumar et al., 2024; Mall et al., 2024; Brant et al., 2025).

Plasmid-based CRISPR systems drive continuous transcription of Cas9 and gRNA throughout regeneration period, leading to persistent DNA scanning across the repeat-dense auto-allopolyploid genome and frequent off-target double-strand breaks (DSBs) on homologous subgenomic paralogs (Ou, 2026; Wang J. et al., 2026). In contrast, pre-assembled RNPs remain biologically active inside cells for only 2–5 days before complete degradation by endogenous nucleases and proteases. This transient activity drastically limits the total number of gRNA-DNA binding events; comparative sugarcane protoplast trials under identical gRNA dosages confirm a significant reduction in detectable off-target variants using RNP delivery compared to plasmid-based systems (Wang Q. et al., 2021; Wu D. et al., 2026). Short-lived RNP exposure also eliminates uneven cleavage across divergent haplotypes, the primary source of mosaic chimerism described. Chimeric sugarcane plants produce inconsistent abiotic stress resistance and fail uniform multi-location field phenotyping trials, while RNP-derived regenerants exhibit uniform indel profiles across homologous gene copies. This consistent genotype substantially cuts labor expenditure and sequencing costs associated with multi-generational whole-genome sequencing (WGS) off-target screening. *Agrobacterium*-mediated and biolistic plasmid delivery often triggers random integration of vector backbones, antibiotic selection markers, and fragmented bacterial sequences into sugarcane chromosomes. These exogenous insertions can disrupt core agronomic regulators, including *SPS*, *WRKY*, *DREB*, and the heavy metal detoxification gene *ScMT2-1-3*, inadvertently impairing sucrose accumulation and stress tolerance phenotypes (Ferreira et al., 2012). DNA-free RNP workflows introduce no foreign DNA into plant cells, eliminating this secondary source of unplanned genomic damage in sugarcane, sugar beet, and sweet sorghum regenerants.

Global agricultural regulatory frameworks draw a definitive distinction between DNA-transgenic lines and transgene-free RNP-edited germplasm. Plasmid-modified sugarcane requires expensive, multi-year ecological and toxicology assessments alongside repeated multi-generational WGS validation to rule out foreign insertions and heritable off-target variants (Liberty et al., 2025). In contrast, transgene-free RNP lines qualify for streamlined approval pathways in China, the EU, the United States, and major sugar-producing nations such as Brazil. This regulatory shortcut shortens the timeline from lab editing to large-scale commercial cultivation by 3–5 years,an invaluable benefit given sugar crops protracted 6–12-month somatic regeneration cycle. Accelerated variety registration speeds the deployment of drought, salinity and heavy metal-tolerant germplasm onto marginal farmlands to sustain stable sucrose and bioethanol production.

### 4.4 Haplotype-specific gRNA design

Haplotype-aware gRNA engineering relies on high-quality haplotype-resolved genome assemblies and sugar crop graph pangenomes to distinguish sequence polymorphisms between divergent paralogous gene haplotypes (Liu et al., 2023; Bao et al., 2024; Healey et al., 2024; Zhang et al., 2025; Huang et al., 2026). Unlike conventional universal gRNAs that bind conserved genomic regions shared across all homologs, the bioinformatic workflow selectively designs guide RNAs that exclusively hybridize to single-nucleotide polymorphisms (SNPs), small insertions or deletions (indels), and haplotype-unique motif signatures unique to one target gene copy (Sternberg and Doudna, 2015). Specialized computational framework, CRISPR-HAWK, demonstrated the feasibility of haplotype-aware gRNA design by integrating individual and population-scale variants into guide selection (Kumbara et al., 2025). However, CRISPR-HAWK was developed for human therapeutic applications using 1000 Genomes and gnomAD datasets, with validation on the human *BCL11A* sickle-cell locus. The conceptual framework is valuable for polyploid sugar crops, direct application to sugarcane require adaptation to incorporate crop-specific pangenome resources and account for the complexities of auto-allopolyploid genomes. Nevertheless, the underlying principle—using haplotype-specific genetic variation to guide gRNA design—remains highly relevant and can inform the development of similar pipelines for polyploid crops as phased pangenome resources become increasingly available. (Sternberg and Doudna, 2015; Huang et al., 2026). Without phased pangenome resources for large polyploid genomes, such as sugarcane, distinguishing haplotype-specific variants from inter-subgenome conserved sequences is computationally unfeasible, making this precision editing strategy inaccessible for unassembled polyploid germplasm (Liu et al., 2023; Pandey et al., 2025; Wang J. et al., 2026).

Haplotype-specific gRNA design enables locus-specific modification of individual gene copies without disrupting functionally valuable homologous haplotypes—a transformative advantage unique to polyploid sugarcane, sugar beet, and sweet sorghum (Liu et al., 2023; Todd et al., 2024; Dohm et al., 2025; Morris et al., 2026; Yanqing et al., 2026). Many elite sugarcane varieties carry coexisting haplotypes with opposing physiological functions: some paralogs negatively regulate abiotic stress tolerance, whereas alternate haplotypes support high sucrose biosynthesis under drought or saline conditions. Traditional non-specific gRNAs cleave every homologous copy simultaneously, abolishing both detrimental and beneficial gene activity and triggering severe pleiotropic yield losses alongside stress improvement. Targeted haplotype gRNAs allow breeders to precisely knockout or mutate only unfavorable stress-suppressing haplotypes while fully preserving intact wild-type alleles responsible for high sugar accumulation. This allele-selective editing eliminates unintended pleiotropic disruptions to sucrose metabolic pathways and avoids irreversible loss of native agronomic haplotypes that underpin commercial variety performance. In downstream breeding, this precision drastically reduces the volume of multi-location field phenotyping required to screen regenerants, as edited lines retain the full complement of favorable native alleles and display stable, predictable stress-resistance phenotypes without compromised biomass or sucrose yield. Additionally, haplotype-specific gRNA cocktails minimize genome-wide off-target DSBs by eliminating broad cross-paralog binding, lowering mandatory multi-generational whole-genome sequencing (WGS) validation costs.

While haplotype-aware gRNA engineering resolves the precision and functional redundancy barriers, it cannot mitigate the core delivery and regeneration bottlenecks. Even perfectly optimized, haplotype-exclusive gRNA sequences are impractical without efficient cellular delivery by biolistic, *Agrobacterium* or RNP workflows; superior guide design does not improve low transformation recovery rates or shorten the somatic regeneration timeline per edited event. Second major constraint stems from polyploid genomic complexity: stress tolerance traits are frequently governed by 3–5 or more functionally redundant haplotypes, each carrying distinct unique variants. Separate custom gRNAs must be synthesized for every target paralog to achieve uniform mutagenesis across all contributing copies, raising reagent costs and requiring complex multiplex transfection setups. Furthermore, post-editing high-depth NGS genotyping remains mandatory to verify complete modification of all intended haplotypes, even with optimized guide design, as variable cellular uptake of distinct gRNA molecules creates partial editing mosaic in polyploid callus. Finally, this strategy is entirely dependent on high-cost phased pangenome sequencing for each commercial sugarcane germplasm; un-sequenced elite varieties lack resolved haplotype variant data, rendering haplotype-specific gRNA design incredible for most non-sequenced breeding material.

### 4.5 Comparative editing strategies for sugar crops

The selection of an appropriate CRISPR strategy to improve abiotic stress tolerance in sugar crops requires careful consideration of several factors, including editing efficiency, heritability, off-target risk, and potential trade-offs with yield and sucrose accumulation rates. Knockout strategies (CRISPR/Cas9 and Cas12a) are the most effective for generating loss-of-function mutations in the negative regulators of stress tolerance. Unlike diploid model species, sugarcane (auto-allopolyploid), and sugar beet (amphidiploid) harbor multiple homoeologous copies of most stress-regulatory genes, meaning that single-gRNA knockouts often fail to elicit phenotypic shifts because unedited paralogs compensate through functional redundancy. However, in polyploid crops, such as sugarcane, functional redundancy across multiple homoeologs necessitates the simultaneous editing of multiple gene copies to achieve a measurable phenotype. For example, the 71 *nsLTP* genes/haplotypes in sugarcane exhibit extensive functional redundancy (Li J. et al., 2022), editing one or two copies often fails to produce significant improvements in membrane stability under drought stress. Successful knockout strategies require custom multiplex gRNA cocktails and high-depth sequencing to verify uniform editing across target copies per regenerated plant, which significantly increases screening costs and breeding timelines. Recent studies demonstrated the multiplex targeting in ​sugarcane, with co-editing of up to 40 copies of target genes, such as *LIGULELESS1 (LG1),* achieved through optimized gRNA design (Brant et al., 2024). Consequently, multiplexed NHEJ targeting all expressed copies is required for knockout strategies in sugarcane. However, this introduces distinct liabilities in repetitive polyploid genomes. The promoter editing approach offers a more nuanced strategy for modulating gene expression without completely abolishing the protein function. Stress-responsive gene expressions can be fine-tuned by introducing targeted mutations in cis-regulatory elements. Recent advances in massively parallel reporter assays in sorghum have demonstrated that compact deletions and targeted motif insertions in core promoter regions can enhance protein production 30-fold relative to that in the wild type (Groover et al., 2026). However, promoter editing requires detailed knowledge of regulatory elements and is technically more challenging than simple knockout approaches.

CRISPR activation (CRISPRa) and interference (CRISPRi) offer compelling alternatives for polyploid sugar crops because can modulate transcription without introducing DNA breaks, thereby circumventing mosaic indel profiles and structural rearrangements (Ding et al., 2022; Srithawong et al., 2026). By recruiting transcriptional effectors (*VP64, VPR*, or *SunTag*) to endogenous promoters, CRISPRa can upregulate stress-protective genes—such as *BvHSF* for heat tolerance or *SsJAZ1* for drought signaling—while CRISPRi can repress negative regulators, such as *SbWRKY50* (Alok et al., 2025). Because these systems do not alter the coding sequence, can reduce the risk of irreversible pleiotropic effects; if a yield penalty emerges, the transcriptional modulation can be reversed by segregating the dCas9 transgene or by employing transient ribonucleoprotein (RNP) activator complexes. Nevertheless, efficacy depends on chromatin accessibility and promoter conservation across haplotypes, which may necessitate haplotype-specific gRNA cocktails (Kumbara et al., 2025).

Base editing and prime editing occupy a precision niche distinct from NHEJ knockouts and transcriptional modulation. Cytosine and adenine base editors achieve direct C→T or A→G substitutions without DSBs, whereas prime editors combine Cas nickase with reverse transcriptase to introduce targeted insertions, deletions, or all types of point mutations via pegRNA guidance (Lin, 2026). In polyploids, these modalities are particularly valuable for engineering cis-regulatory SNPs or correcting dominant-negative variants without the collateral damage caused by multiplex NHEJ. Successful prime editing in hexaploid wheat, rice and potato demonstrates the feasibility of precise polyploid modification (Lin et al., 2020; Lin, 2026), although neither technology has yet been reported in sugarcane. A critical limitation is that each haplotype must be individually targeted if variants differ at the edit site, requiring high-depth amplicon sequencing to confirm uniform modifications across paralogs.​

### 4.6 Polyploid constraints: Mosaics, partial alteration, and structural variation

Genotype-dependent regeneration is the primary translational bottleneck. Elite commercial sugarcane varieties display profound recalcitrance toward *Agrobacterium*-mediated transformation and biolistic bombardment. This barrier is genotype-dependent and driven by ploidy-triggered epigenetic silencing of exogenous DNA or RNPs, as well as tissue-specific cell wall rigidity, which impedes vector penetration during transfection (Antony Ceasar and Ignacimuthu, 2023; Altpeter, 2025). The resulting somatic regeneration cycle per editing event far exceeds diploid models such as rice or *Arabidopsis*, drastically inflating labor costs and delaying germplasm screening (Brant et al., 2024; Altpeter, 2025; Brant et al., 2025; Wang W. et al., 2026). Editing mosaics and partial alteration of homologous copies are inevitable consequences of polyploidy. In sugarcane callus, uneven cleavage across divergent haplotypes produces chimeric plants. This mosaicism is invisible in shallow amplicon sequencing and is only revealed by high-depth long-read genotyping or phenotypic segregation in ratoon crops. Partial alteration of 1–2 copies in a 4x or 8x background typically yields no phenotypic shift due to dosage compensation; breeders must verify uniform editing across at least three to five paralogous copies per regenerated plant to breach the functional redundancy threshold. Additionally, capillary electrophoresis provides a cost-effective alternative to next-generation sequencing for detecting indel mosaicism in polyploid sugarcane (Eid et al., 2021; Altpeter, 2025; Brant et al., 2025; Li et al., 2026).

On-target structural changes represent a severe but under a appreciated risk of multiplexed NHEJ in polyploid genomes. Simultaneous DSBs at adjacent target sites can generate large chromosomal deletions, inversions, and translocations between homoeologous chromosomes (Sturme et al., 2022). In poplar, multiplex editing of seven closely linked *NRX1* genes induced diverse mutations, including translocations and inversions (Feng et al., 2025). In sugarcane’s ∼10 Gb auto-allopolyploid genome, harbors numerous segmental duplications and repetitive intergenic sequences, such structural variants can inadvertently disrupt sucrose phosphate synthase (*SPS*), *WRKY*, or *DREB* loci, negating the primary economic trait. Base and prime editing, which operate without DSBs, largely circumvent risk and should be prioritized when the breeding objective is a precise nucleotide change rather than gene elimination (Li et al., 2023; Wang T. et al., 2026).

### 4.7 Pleiotropy, yield penalty and dosage sensitivity

A critical but often overlooked consideration is the coupling of stress tolerance with yield or sucrose penalties. Stress-regulatory transcription factors are frequently embedded in hormones and metabolic networks that directly influence carbon partitioning. Constitutive overexpression of *DREB1A* confers drought, salt, and freezing tolerance but reduces plant growth, development, and reproduction by diverting carbon skeletons from biomass accumulation (Kasuga et al., 1999). Similarly, the constitutive activation of *WRKY* or *HSF* pathways may enhance antioxidant defense but impose a Brix penalty under non-stress conditions by repressing growth-promoting genes or exhausting cellular energy reserves.

Recent advances in promoter editing offer a route to decouple these traits. In maize, CRISPR-mediated insertion of drought-responsive cis-regulatory elements into the *ARGOS8* promoter increased yield by up to five bushels per acre under drought without reducing yield under normal irrigation, demonstrating that tissue-specific or stress-inducible transcriptional tuning can improve resilience without an agronomic penalty (Shi et al., 2017). For sugar crops, analogous strategies—such as root-specific CRISPRa of *BvDREB* or stress-inducible activation of *ScNsLTP* promoters—may allow abiotic stress tolerance to be uncoupled from stalk sucrose content, though this hypothesis remains experimentally untested.

### 4.8 Field validation and heritability

Despite rapid progress in target identification and *in vitro* editing, no CRISPR-edited sugar crop has progressed to multi-location field trials for abiotic stress tolerance so far. Although field-validated CRISPR-edited maize (*ZmHDT103*) and rice (*OsRR22*) have demonstrated heritable stress resistance under multi-environment (Gao, 2021; Wang et al., 2024), equivalent validation in vegetatively propagated polyploid sugar crops is lacking. The sugarcane regeneration cycle, combined with ratoon propagation, needs to verify mitotic stability, that heredity of edited alleles remains uncharacterized in commercial germplasm. RNP-derived edits must be confirmed as stable through at least two vegetative generations, and stress phenotypes must be validated under field abiotic stress rather than *in vitro* or greenhouse conditions. Furthermore, global regulatory frameworks categorize heritable off-target variants as uncharacterized genomic alterations, necessitating expensive multi-generational whole-genome sequencing validation before variety registration (Liberty et al., 2025). Until field data and multi-generational inheritance studies are available, claims of “enhanced tolerance” in sugar crops remain pre-commercial hypotheses requiring rigorous phenotypic validation.

## 5 Conclusion and future prospectives

CRISPR-based systems offer substantial advantages over other genome-editing technologies because of their precision and user-friendly design. Recent achievements in targeting multiple gene copies in sugarcane underscore the feasibility of genome editing in polyploid crops, facilitating the development of varieties with improved tolerance to diverse abiotic and biotic stresses. CRISPR/Cas tools have significant potential for developing sugar beet breeding lines that exhibit resistance or tolerance to various abiotic and biotic stressors. By silencing or knocking out specific genes using CRISPR/Cas9/12, prime editors, and base editors, sugar beets can be engineered to adapt to challenging environmental conditions, thereby enhancing their physiological and biochemical stress tolerance. Furthermore, CRISPR-based methodologies enable the creation of novel traits via gain-of-function mutations, thereby contributing to a more comprehensive understanding of the gene regulatory networks involved in stress responses and supporting efforts to improve crops.

The application of CRISPR-based systems in sugarcane cultivation represents a significant advancement in agricultural biotechnology. Recent advancements in sugar crop genomics (e.g., reference genome availability, haplotype-resolved assemblies, and CRISPR/Cas reagent optimization- (Supplementary Table S1) have further enhanced its feasibility. These tools address longstanding challenges in sugar crop improvement under abiotic stress, offering a path toward sustainable, precision-bred varieties that maintain yield and quality even in adverse environmental conditions, meeting consumer and industrial demands. Furthermore, genome-editing research must address several challenges, including gene delivery issues, the risk of off-target effects, and the limited efficacy of current techniques. Despite these challenges, such research opens new avenues for sugar crop genome editing, potentially leading to a more resilient, productive, and sustainable agricultural future for sugar crops.

## References

[B1] Abdel-Ghany S. E. Ullah F. Ben-Hur A. Reddy A. S. N. (2020). Transcriptome analysis of drought-resistant and drought-sensitive sorghum (*Sorghum bicolor*) genotypes in response to PEG-induced drought stress. IJMS 21, 772. 10.3390/ijms21030772 31991584 PMC7037816

[B2] Abu-Ellail F. F. B. Hussein E. M. A. Attafy T. M. (2024). Categorization of sugar beet varieties for water saving in sandy soils using factor analysis scores. Sugar Tech. 26, 1732–1745. 10.1007/s12355-024-01359-3

[B3] Abu-Ellail F. F. B. Bassiony N. A. M. Abdelkawy R. N. F. (2025). Using multivariate analyses to evaluate the impact of proline on sugar and root yield traits in sugar beet under saline conditions. Bull. Natl. Res. Centre 49, 46. 10.1186/s42269-025-01338-y

[B4] Alok A. Chauhan H. Rout B. Pandey A. Singh K. (2025). CRISPR/dCas9-KRAB mediated transcriptional suppression of *NtbHLH47* enhances tolerance to iron stress and modulates iron content in tobacco. Plant Sci. 354, 112449. 10.1016/j.plantsci.2025.112449 40015341

[B5] Altpeter F. (2025). Gene editing to enhance biotic stress tolerance in sugarcane. Front. Plant Sci. 16, 1750169. 10.3389/fpls.2025.1750169 PMC12785182 41523156

[B6] Ameen M. Mahmood A. Shahzad A. N. Zia M. A. Javaid M. M. (2024). Sorghum’s potential unleashed: a comprehensive exploration of bio-energy production strategies and innovations. Bioresour. Technol. Rep. 27, 101906. 10.1016/j.biteb.2024.101906

[B7] Antony Ceasar S. Ignacimuthu S. (2023). CRISPR/cas genome editing in plants: dawn of *agrobacterium* transformation for recalcitrant and transgene-free plants for future crop breeding. Plant Physiology Biochem. 196, 724–730. 10.1016/j.plaphy.2023.02.030 36812799

[B8] Appiah-Nkansah N. B. Li J. Rooney W. Wang D. (2019). A review of sweet sorghum as a viable renewable bioenergy crop and its techno-economic analysis. Renew. Energy 143, 1121–1132. 10.1016/j.renene.2019.05.066

[B9] Bao Y. Zhang Q. Huang J. Zhang S. Yao W. Yu Z. (2024). A chromosomal-scale genome assembly of modern cultivated hybrid sugarcane provides insights into origination and evolution. Nat. Commun. 15, 3041. 10.1038/s41467-024-47390-6 38589412 PMC11001919

[B10] Begcy K. Mariano E. D. Lembke C. G. Zingaretti S. M. Souza G. M. Araújo P. (2019). Overexpression of an evolutionarily conserved drought-responsive sugarcane gene enhances salinity and drought resilience. Ann. Bot. 124, 691–700. 10.1093/aob/mcz044 31125059 PMC6821327

[B11] Ben-Tov D. Mafessoni F. Cucuy A. Honig A. Melamed-Bessudo C. Levy A. A. (2024). Uncovering the dynamics of precise repair at CRISPR/Cas9-induced double-strand breaks. Nat. Commun. 15, 5096. 10.1038/s41467-024-49410-x 38877047 PMC11178868

[B12] Birch R. G. Bower R. S. Elliott A. R. (2010). Highly efficient, 5′-Sequence-Specific transgene silencing in a complex polyploid. Trop. Plant Biol. 3, 88–97. 10.1007/s12042-010-9047-0

[B13] Bozdag G. O. Kaya A. Koc A. Noll G. A. Prüfer D. Karakaya H. C. (2014). Characterization of a cDNA from *Beta maritima* that confers nickel tolerance in yeast. Gene 538, 251–257. 10.1016/j.gene.2014.01.052 24487090

[B14] Brant E. J. Eid A. Kannan B. Baloglu M. C. Altpeter F. (2024). The extent of multiallelic, co‐editing of *LIGULELESS1* in highly polyploid sugarcane tunes leaf inclination angle and enables selection of the ideotype for biomass yield. Plant Biotechnol. J. 22, 2660–2671. 10.1111/pbi.14380 38776398 PMC11536458

[B15] Brant E. Zuniga‐Soto E. Altpeter F. (2025). RNAi and genome editing of sugarcane: progress and prospects. Plant J. 121, e70048. 10.1111/tpj.70048 40051334 PMC11886501

[B16] Carnavale Bottino M. Rosario S. Grativol C. Thiebaut F. Rojas C. A. Farrineli L. (2013). High-throughput sequencing of small RNA transcriptome reveals salt stress regulated MicroRNAs in sugarcane. PLoS ONE 8, e59423. 10.1371/journal.pone.0059423 23544066 PMC3609749

[B17] Chan F. K. S. Chen J. Johnson M. F. Shao Y. Wang J. Wang J. (2025). Food security under climatic extremes in the Asia-Pacific region. Npj Clim. Action 4, 111. 10.1038/s44168-025-00316-4

[B18] Chen Y. Ma J. Zhang X. Yang Y. Zhou D. Yu Q. (2017). A novel non-specific lipid transfer protein gene from sugarcane (*NsLTPs*), obviously responded to abiotic stresses and signaling molecules of SA and MeJA. Sugar Tech. 19, 17–25. 10.1007/s12355-016-0431-4

[B19] Chen C. Shang X. Sun M. Tang S. Khan A. Zhang D. (2022). Comparative transcriptome analysis of two sweet sorghum genotypes with different salt tolerance abilities to reveal the mechanism of salt tolerance. IJMS 23, 2272. 10.3390/ijms23042272 35216389 PMC8877675

[B20] Cheng Z. Li K. Liu H. Wei X. Yin T. Xing X. (2024). Establishment of a genome‐editing system to create fragrant germplasm in sweet sorghum. aBIOTECH 5, 502–506. 10.1007/s42994-024-00180-6 39650140 PMC11624148

[B21] Chopra R. Burow G. Hayes C. Emendack Y. Xin Z. Burke J. (2015). Transcriptome profiling and validation of gene based single nucleotide polymorphisms (SNPs) in sorghum genotypes with contrasting responses to cold stress. BMC Genomics 16, 1040. 10.1186/s12864-015-2268-8 26645959 PMC4673766

[B22] Cossio L. A. Melgar A. E. Moyano L. Schrauf G. E. Zelada A. M. (2025). Genome-wide identification of lipid transfer proteins in *Sorghum bicolor* and discovery of flower-specific promoters. Sci. Rep. 15, 25710. 10.1038/s41598-025-08625-8 40670441 PMC12267850

[B23] Cui J. Sun Z. Li J. Cheng D. Luo C. Dai C. (2018). Characterization of miRNA160/164 and their targets expression of beet (*beta vulgaris*) seedlings under the salt tolerance. Plant Mol. Biol. Rep. 36, 790–799. 10.1007/s11105-018-1118-7

[B24] Ding X. Yu L. Chen L. Li Y. Zhang J. Sheng H. (2022). Recent progress and future prospect of CRISPR/Cas-Derived transcription activation (CRISPRa) System in plants. Cells 11, 3045. 10.3390/cells11193045 36231007 PMC9564188

[B25] Dohm J. C. Holzweber T. Pensch R. A. Himmelbauer H. (2025). Sugar beet long-read reference assembly of genotype KWS2320. Nar. Genom Bioinform 7, lqaf142. 10.1093/nargab/lqaf142 41244989 PMC12614215

[B26] Eid A. Mohan C. Sanchez S. Wang D. Altpeter F. (2021). Multiallelic, targeted mutagenesis of magnesium chelatase with CRISPR/Cas9 provides a rapidly scorable phenotype in highly polyploid sugarcane. Front. Genome Ed. 3, 654996. 10.3389/fgeed.2021.654996 34713257 PMC8525377

[B27] Erbasol I. Bozdag G. O. Koc A. Pedas P. Karakaya H. C. (2013). Characterization of two genes encoding metal tolerance proteins from *Beta vulgaris* subspecies maritima that confers manganese tolerance in yeast. Biometals 26, 795–804. 10.1007/s10534-013-9658-7 23864431

[B28] Feng Y. Sun Y. Zhang H. Guo X. Feng Y. Liu D. (2025). Increased genome editing efficiency in poplar by optimizing sgRNA length and copy number. Industrial Crops Prod. 226, 120664. 10.1016/j.indcrop.2025.120664

[B29] Ferreira T. H. Gentile A. Vilela R. D. Costa G. G. L. Dias L. I. Endres L. (2012). microRNAs associated with drought response in the bioenergy crop sugarcane (*Saccharum* spp.). PLoS ONE 7, e46703. 10.1371/journal.pone.0046703 23071617 PMC3469577

[B30] Gao C. (2021). Genome engineering for crop improvement and future agriculture. Cell 184, 1621–1635. 10.1016/j.cell.2021.01.005 33581057

[B31] Ghuge S. A. Nikalje G. C. Kadam U. S. Suprasanna P. Hong J. C. (2023). Comprehensive mechanisms of heavy metal toxicity in plants, detoxification, and remediation. J. Hazard. Mater. 450, 131039. 10.1016/j.jhazmat.2023.131039 36867909

[B32] Giri B. Kumar D. (2026). Plant non-specific lipid transfer proteins (nsLTPs): comprehensive functional analysis and defense mechanisms. Biology 15, 417. 10.3390/biology15050417 41823844 PMC12985213

[B33] Groover E. D. Ding D. Wang F. Z. Benegas G. Rivera J. Schwartz S. (2026). Mapping cis-regulatory mutations at scale in sorghum enables modulation of gene expression. Nat. Biotechnol. 1–11. 10.1038/s41587-026-03046-y 41896479

[B34] Guan K. Yang Z. Zhan M. Zheng M. You J. Meng X. (2023). Two sweet sorghum (*Sorghum bicolor* L.) WRKY transcription factors promote aluminum tolerance *via* the reduction in callose deposition. IJMS 24, 10288. 10.3390/ijms241210288 PMC10298921 37373435

[B35] Guo J. Xu L. Su Y. Wang H. Gao S. Xu J. (2013). *ScMT2-1-3*, a metallothionein gene of sugarcane, plays an important role in the regulation of heavy metal tolerance/accumulation. BioMed Res. Int. 2013, 1–12. 10.1155/2013/904769 23781509 PMC3677015

[B36] Guo J. Ling H. Ma J. Chen Y. Su Y. Lin Q. (2017). A sugarcane R2R3-MYB transcription factor gene is alternatively spliced during drought stress. Sci. Rep. 7, 41922. 10.1038/srep41922 28167824 PMC5294458

[B37] Guo Y. Y. Tian S. S. Liu S. S. Wang W. Q. Sui N. (2018). Energy dissipation and antioxidant enzyme system protect photosystem II of sweet sorghum under drought stress. Photosynt 56, 861–872. 10.1007/s11099-017-0741-0

[B38] Haque A. M. Tasnim J. El-Shehawi A. M. Rahman M. A. Parvez M. S. Ahmed M. B. (2021). The Cd-induced morphological and photosynthetic disruption is related to the reduced Fe status and increased oxidative injuries in sugar beet. Plant Physiology Biochem. 166, 448–458. 10.1016/j.plaphy.2021.06.020 34161881

[B39] Healey A. L. Garsmeur O. Lovell J. T. Shengquiang S. Sreedasyam A. Jenkins J. (2024). The complex polyploid genome architecture of sugarcane. Nature 628, 1–7. 10.1038/s41586-024-07231-4 PMC11041754 38538783

[B40] Hossain Md. S. Islam Md. N. Rahman Md. M. Mostofa M. G. Khan Md. A. R. (2022). Sorghum: a prospective crop for climatic vulnerability, food and nutritional security. J. Agric. Food Res. 8, 100300. 10.1016/j.jafr.2022.100300

[B41] Houmani H. Corpas F. J. (2026). Potassium: a master regulator of abiotic stress mitigation and synergistic enhancer of plant resilience. J. Crop Sci. Biotechnol. 29, 355–370. 10.1007/s12892-026-00352-4

[B42] Huang Y. Zhang Y. Zhang Q. Zhuang G. Li C. Wang B. (2026). Multiscale pangenome graphs empower the genomic dissection of mixed-ploidy sugarcane species. Science 391, eadx1616. 10.1126/science.adx1616 41643009

[B43] Hui Q. Song T. Yang D. Wu Q. Guo J. Que Y. (2024). Identification and characterization of key genes for nitrogen utilization from *Saccharum spontaneum* sub-genome in modern sugarcane cultivar. IJMS 26, 226. 10.3390/ijms26010226 39796079 PMC11720480

[B44] Hussain H. A. Hussain S. Khaliq A. Ashraf U. Anjum S. A. Men S. (2018). Chilling and drought stresses in crop plants: implications, cross talk, and potential management opportunities. Front. Plant Sci. 9, 393. 10.3389/fpls.2018.00393 29692787 PMC5902779

[B45] Hussain A. Ding X. Alariqi M. Manghwar H. Hui F. Li Y. (2021). Herbicide resistance: another hot agronomic trait for plant genome editing. Plants 10, 621. 10.3390/plants10040621 33805182 PMC8064318

[B46] Ismail R. Youssef A. El-Assal S. Tawfik M. Abdallah N. (2020). Cloning and characterization oof heat shock factor (*Bvhsf*) from sugar beet (*Beta vulgaris*). Available online at: https://www.semanticscholar.org/paper/ (Accessed March 31, 2026).

[B47] Jia W. Guo Z. Lv S. Lin K. Li Y. (2024). SbYS1 and SbWRKY72 regulate Cd tolerance and accumulation in sweet sorghum. Planta 259, 100. 10.1007/s00425-024-04388-0 38536457

[B48] Jiang J. Ma S. Ye N. Jiang M. Cao J. Zhang J. (2017). *WRKY* transcription factors in plant responses to stresses. JIPB 59, 86–101. 10.1111/jipb.12513 27995748

[B49] Jin-long G. Li-ping X. Jing-ping F. Ya-chun S. Hua-ying F. You-xiong Q. (2012). A novel dirigent protein gene with highly stem-specific expression from sugarcane, response to drought, salt and oxidative stresses. Plant Cell Rep. 31, 1801–1812. 10.1007/s00299-012-1293-1 22696141

[B50] Kasuga M. Liu Q. Miura S. Yamaguchi-Shinozaki K. Shinozaki K. (1999). Improving plant drought, salt, and freezing tolerance by gene transfer of a single stress-inducible transcription factor. Nat. Biotechnol. 17, 287–291. 10.1038/7036 10096298

[B51] Keller I. Müdsam C. Rodrigues C. M. Kischka D. Zierer W. Sonnewald U. (2021). Cold-Triggered induction of ROS- and raffinose metabolism in freezing-sensitive taproot tissue of sugar beet. Front. Plant Sci. 12, 715767. 10.3389/fpls.2021.715767 34539707 PMC8446674

[B52] Kito K. Yamane K. Yamamori T. Matsuhira H. Tanaka Y. Takabe T. (2017). Isolation, functional characterization and stress responses of raffinose synthase genes in sugar beet. J. Plant Biochem. Biotechnol. 27, 36–45. 10.1007/s13562-017-0413-y

[B53] Kristensen A. K. Brunstedt J. Nielsen K. K. Roepstorff P. Mikkelsen J. D. (2000). Characterization of a new antifungal non-specific lipid transfer protein (nsLTP) from sugar beet leaves. Plant Sci. 155, 31–40. 10.1016/S0168-9452(00)00190-4 10773337

[B54] Kumar T. Uzma Khan M. R. Abbas Z. Ali G. M. (2014). Genetic improvement of sugarcane for drought and salinity stress tolerance using arabidopsis vacuolar pyrophosphatase (*AVP1*) gene. Mol. Biotechnol. 56, 199–209. 10.1007/s12033-013-9695-z 24002911

[B55] Kumar T. Bao A.-K. Bao Z. Wang F. Gao L. Wang S.-M. (2018). The progress of genetic improvement in alfalfa (*Medicago sativa* L.). Czech J. Genet. Plant Breed. 54, 41–51. 10.17221/46/2017-CJGPB

[B56] Kumar T. Wang J.-G. Xu C.-H. Lu X. Mao J. Lin X.-Q. (2024). Genetic engineering for enhancing sugarcane tolerance to biotic and abiotic stresses. Plants 13, 1739. 10.3390/plants13131739 38999579 PMC11244436

[B57] Kumbara A. Tognon M. Carone G. Fontanesi A. Bombieri N. Giugno R. (2025). CRISPR-HAWK: Haplotype- and Variant-aware Guide Design Toolkit for CRISPR-Cas. 10.64898/2025.12.27.696698 PMC13674355 42872486

[B58] Lei G. Shen M. Li Z. Zhang B. Duan K. Wang N. (2011). EIN2 regulates salt stress response and interacts with a MA3 domain‐containing protein ECIP1 in *Arabidopsis* . Plant Cell and Environ. 34, 1678–1692. 10.1111/j.1365-3040.2011.02363.x 21631530

[B59] Li C. Iqbal M. A. (2024). Leveraging the sugarcane CRISPR/Cas9 technique for genetic improvement of non-cultivated grasses. Front. Plant Sci. 15, 1369416. 10.3389/fpls.2024.1369416 38601306 PMC11004347

[B60] Li J. Phan T.-T. Li Y.-R. Xing Y.-X. Yang L.-T. (2018). Isolation, transformation and overexpression of sugarcane *SoP5CS* gene for drought tolerance improvement. Sugar Tech. 20, 464–473. 10.1007/s12355-017-0568-9

[B61] Li J. Cui J. Dai C. Liu T. Cheng D. Luo C. (2020). Whole-transcriptome RNA sequencing reveals the global molecular responses and CeRNA regulatory network of mRNAs, lncRNAs, miRNAs and circRNAs in response to salt stress in sugar beet (*Beta vulgaris*). IJMS 22, 289. 10.3390/ijms22010289 33396637 PMC7795855

[B62] Li J. Zhao J.-Y. Shi Y. Fu H.-Y. Huang M.-T. Meng J.-Y. (2022a). Systematic and functional analysis of non-specific lipid transfer protein family genes in sugarcane under *Xanthomonas albilineans* infection and salicylic acid treatment. Front. Plant Sci. 13, 1014266. 10.3389/fpls.2022.1014266 PMC9581186 36275567

[B63] Li Z. Tong Z. He F. Li X. Sun J. (2022b). Integrated mRNA and microRNA expression analysis of root response to phosphate deficiency in *Medicago sativa* . Front. Plant Sci. 13, 989048. 10.3389/fpls.2022.989048 PMC9513243 36176687

[B64] Li J. Zhang C. He Y. Li S. Yan L. Li Y. (2023). Plant base editing and prime editing: the current status and future perspectives. JIPB 65, 444–467. 10.1111/jipb.13425 36479615

[B65] Li C. Li X. Tian C. Hu X. Lu X. Nawaz G. (2026). Establishment of an efficient *agrobacterium*-mediated genetic transformation protocol for *Saccharum officinarum* using Black Cheribon as a model genotype. Front. Plant Sci. 17, 1893606. 10.3389/fpls.2026.1893606 42712694 PMC13550202

[B66] Liberty J. T. Poudel B. Ihedioha O. Lin H. Habanabakize E. Gao Z. (2025). Gene editing technology: shaping international standards for health and food safety assurance. Trends Biotechnol. 43, 985–988. 10.1016/j.tibtech.2024.09.019 39424445

[B67] Lin Q. (2026). Prime editing: redefining precision genome editing. Nat. Rev. Genet. 27, 583. 10.1038/s41576-026-00965-z 42086924

[B68] Lin Q. Zong Y. Xue C. Wang S. Jin S. Zhu Z. (2020). Prime genome editing in rice and wheat. Nat. Biotechnol. 38, 582–585. 10.1038/s41587-020-0455-x 32393904

[B69] Liu F. Zhang X. Lu C. Zeng X. Li Y. Fu D. (2015). Non-specific lipid transfer proteins in plants: presenting new advances and an integrated functional analysis. EXBOTJ 66, 5663–5681. 10.1093/jxb/erv313 26139823

[B70] Liu F. Huang N. Wang L. Ling H. Sun T. Ahmad W. (2018). A novel L-ascorbate peroxidase 6 gene, ScAPX6, plays an important role in the regulation of response to biotic and abiotic stresses in sugarcane. Front. Plant Sci. 8, 2262. 10.3389/fpls.2017.02262 29387074 PMC5776131

[B71] Liu H. Lin X. Li X. Luo Z. Lu X. You Q. (2023). Haplotype variations of sucrose phosphate synthase B gene among sugarcane accessions with different sucrose content. BMC Genomics 24, 42. 10.1186/s12864-023-09139-1 36698074 PMC9875459

[B72] Liu H.-B. Kumar T. Lin X.-Q. Xu C.-H. Mao J. Kong C.-Y. (2026). Integrated metabolomics and transcriptomics analysis of exogenous arginine-mediated sucrose accumulation in sugarcane. IJMS 27, 5476. 10.3390/ijms27125476 42353192 PMC13299503

[B73] Mall A. K. Manimekalai R. Misra V. Pandey H. Srivastava S. Sharma A. (2024). CRISPR/Cas-Mediated genome editing for sugarcane improvement. Sugar Tech. 27, 1–13. 10.1007/s12355-023-01352-2

[B74] Marla S. R. Shiva S. Welti R. Liu S. Burke J. J. Morris G. P. (2017). Comparative transcriptome and lipidome analyses reveal molecular chilling responses in chilling‐tolerant sorghums. Plant Genome 10 (3). 10.3835/plantgenome2017.03.0025 29293808

[B75] Matsuhira H. Shinada H. Yui-Kurino R. Hamato N. Umeda M. Mikami T. (2007). An anther-specific lipid transfer protein gene in sugar beet: its expression is strongly reduced in male-sterile plants with owen cytoplasm. Physiol. Plant. 129, 407–414. 10.1111/j.1399-3054.2006.00813.x

[B76] Maulana F. Tesso T. T. (2013). Cold temperature episode at seedling and flowering stages reduces growth and yield components in sorghum. Crop Sci. 53, 564–574. 10.2135/cropsci2011.12.0649

[B77] Misra V. Mall A. K. Pandey H. Srivastava S. Sharma A. (2023). Advancements and prospects of CRISPR/Cas9 technologies for abiotic and biotic stresses in sugar beet. Front. Genet. 14, 1235855. 10.3389/fgene.2023.1235855 38028586 PMC10665535

[B78] Mittler R. Zandalinas S. I. Fichman Y. Van Breusegem F. (2022). Reactive oxygen species signalling in plant stress responses. Nat. Rev. Mol. Cell Biol. 23, 663–679. 10.1038/s41580-022-00499-2 35760900

[B79] Morris G. P. Harder A. M. Healey A. L. McLaughlin C. M. Rifkin J. L. Cruet-Burgos C. (2026). A sorghum pangenome reference improves global crop trait discovery. Nature 652, 1245–1253. 10.1038/s41586-026-10229-9 41813899 PMC13128447

[B80] Mulaudzi-Masuku T. Mutepe R. D. Mukhoro O. C. Faro A. Ndimba B. (2015). Identification and characterization of a heat-inducible *Hsp70* gene from Sorghum bicolor which confers tolerance to thermal stress. Cell Stress Chaperones 20, 793–804. 10.1007/s12192-015-0591-2 26072391 PMC4529866

[B81] Murugan N. Kumar R. Pandey S. Dhansu P. Chennappa M. Nallusamy S. (2023). *In silico* dissection of regulatory regions of PHT genes from saccharum spp. hybrid and *Sorghum bicolor* and expression analysis of PHT promoters under osmotic stress conditions in tobacco. Sustainability 15, 1048. 10.3390/su15021048

[B82] Nagaraju M. Kumar S. A. Reddy P. S. Kumar A. Rao D. M. Kavi Kishor P. B. (2019). Genome-scale identification, classification, and tissue specific expression analysis of late embryogenesis abundant (LEA) genes under abiotic stress conditions in Sorghum bicolor L. PLoS ONE 14, e0209980. 10.1371/journal.pone.0209980 30650107 PMC6335061

[B83] Namata M. J. Xu J. Habyarimana E. Palakolanu S. R. Wang L. Li J. (2025). Genome editing in maize and sorghum: a comprehensive review of CRISPR/Cas9 and emerging technologies. Plant Genome 18, e70038. 10.1002/tpg2.70038 40324959 PMC12052613

[B84] Nap J.-P. de Ruijter F. J. van Es D. S. van der Meer I. M. (2025). The case of sugar beet in Europe: a review of the challenges for a traditional food crop on the verge of climate change and circular agriculture. J. Agric. Food Res. 24, 102343. 10.1016/j.jafr.2025.102343

[B85] Osakabe Y. Watanabe T. Sugano S. S. Ueta R. Ishihara R. Shinozaki K. (2016). Optimization of CRISPR/Cas9 genome editing to modify abiotic stress responses in plants. Sci. Rep. 6, 26685. 10.1038/srep26685 27226176 PMC4880914

[B86] Ou S. (2026). New tools crack repetitive cane. Nat. Plants 12, 1–2. 10.1038/s41477-026-02337-1 42337343

[B87] Pacesa M. Pelea O. Jinek M. (2024). Past, present, and future of CRISPR genome editing technologies. Cell 187, 1076–1100. 10.1016/j.cell.2024.01.042 38428389

[B88] Pandey H. Misra V. Mall A. K. Sharma A. Edwin Hillary V. Ceasar S. A. (2025). Enhancing sugar crop resilience to abiotic stress using CRISPR/Cas tools. Sugar Tech. 27, 985–1002. 10.1007/s12355-025-01561-x

[B89] Park J.-W. Benatti T. R. Marconi T. Yu Q. Solis-Gracia N. Mora V. (2015). Cold responsive gene expression profiling of sugarcane and *Saccharum spontaneum* with functional analysis of a cold inducible saccharum homolog of NOD26-Like intrinsic protein to salt and water stress. PLoS ONE 10, e0125810. 10.1371/journal.pone.0125810 25938773 PMC4418668

[B90] Prabu G. Theertha Prasad D. (2012). Functional characterization of sugarcane *MYB* transcription factor gene promoter (PScMYBAS1) in response to abiotic stresses and hormones. Plant Cell Rep. 31, 661–669. 10.1007/s00299-011-1183-y 22083650

[B91] Qian Z. Zhao C. Wan H. Rao X. Luo Z. He L. (2025). Genome-wide identification of DREB1 transcription factors in Erianthus fulvus and the functional role of *EfDREB1C* in regulating cold tolerance of transgenic Arabidopsis and sugarcane. Int. J. Biol. Macromol. 317, 144859. 10.1016/j.ijbiomac.2025.144859 40460955

[B92] Qiande M. Altpeter F. (2025). Comparing four heat-inducible promoters in stably transformed sugarcane regarding spatial and temporal control of transgene expression reveals candidates to drive stem-preferred transgene expression. Front. Plant Sci. 16, 1709171. 10.3389/fpls.2025.1709171 41415659 PMC12708596

[B93] Qiu W. Li H. Song Y. Ding J. Chen S. Ma C. (2024). Genome-Wide analysis of DREB genes in sugar beet and their potential functions in response to drought stress. Sugar Tech. 26, 1306–1322. 10.1007/s12355-023-01337-1

[B94] Rao Y. Peng T. Xue S. (2023). Mechanisms of plant saline-alkaline tolerance. J. Plant Physiology 281, 153916. 10.1016/j.jplph.2023.153916 36645936

[B95] Reyer A. Bazihizina N. Jaślan J. Scherzer S. Schäfer N. Jaślan D. (2024). Sugar beet PMT5a and STP13 carriers suitable for proton‐driven plasma membrane sucrose and glucose import in taproots. Plant J. 118, 2219–2232. 10.1111/tpj.16740 38602250

[B96] Shi J. Gao H. Wang H. Lafitte H. R. Archibald R. L. Yang M. (2017). *ARGOS 8* variants generated by CRISPR ‐cas9 improve maize grain yield under field drought stress conditions. Plant Biotechnol. J. 15, 207–216. 10.1111/pbi.12603 27442592 PMC5258859

[B97] Song Y. Li J. Sui Y. Han G. Zhang Y. Guo S. (2020). The sweet sorghum *SbWRKY50* is negatively involved in salt response by regulating ion homeostasis. Plant Mol. Biol. 102, 603–614. 10.1007/s11103-020-00966-4 32052233

[B98] Sperdouli I. (2022). Heavy metal toxicity effects on plants. Toxics 10, 715. 10.3390/toxics10120715 36548548 PMC9788363

[B99] Srithawong S. Fang W. Jing Y. Pholtaisong J. Li D. Khumla N. (2026). Sugarcane breeding in the genomic era: integrative strategies and emerging technologies. Plants 15, 286. 10.3390/plants15020286 41600093 PMC12844685

[B100] Sternberg S. H. Doudna J. A. (2015). Expanding the biologist’s toolkit with CRISPR-Cas9. Mol. Cell 58, 568–574. 10.1016/j.molcel.2015.02.032 26000842

[B101] Stracke R. Holtgräwe D. Schneider J. Pucker B. Rosleff Sörensen T. Weisshaar B. (2014). Genome-wide identification and characterisation of R2R3-MYB genes in sugar beet (*Beta vulgaris*). BMC Plant Biol. 14, 249. 10.1186/s12870-014-0249-8 25249410 PMC4180131

[B102] Sturme M. H. J. Van Der Berg J. P. Bouwman L. M. S. De Schrijver A. De Maagd R. A. Kleter G. A. (2022). Occurrence and Nature of off-target modifications by CRISPR-cas genome editing in plants. ACS Agric. Sci. Technol. 2, 192–201. 10.1021/acsagscitech.1c00270 35548699 PMC9075866

[B103] Su Y. Wang Z. Liu F. Li Z. Peng Q. Guo J. (2016). Isolation and characterization of ScGluD2, a new sugarcane beta-1,3-Glucanase D family gene induced by *Sporisorium scitamineum*, ABA, H2O2, NaCl, and CdCl_2_ stresses. Front. Plant Sci. 7, 1348. 10.3389/fpls.2016.01348 27642288 PMC5009122

[B104] Sui N. Yang Z. Liu M. Wang B. (2015). Identification and transcriptomic profiling of genes involved in increasing sugar content during salt stress in sweet sorghum leaves. BMC Genomics 16, 534. 10.1186/s12864-015-1760-5 26186930 PMC4506618

[B105] Sun Y. Li C. Li Z. Jian C. Li N. Zhang S. (2024). Construction of gene expression profiles of the NAC transcription factor family in sugar beet under abiotic stress. Sugar Tech. 26, 1232–1242. 10.1007/s12355-024-01436-7

[B106] Tirabante Terrones N. Gonçalves V. R. Nelissen H. Gonzalez N. Souza Bernardes W. Faleiro De Lima R. (2026). Enhanced expression of the *ScTpx2* gene confers tolerance to drought stress in transgenic sugarcane. GM Crops and Food 17, 2612426. 10.1080/21645698.2025.2612426 41670019 PMC12915768

[B107] Tiroli A. O. Ramos C. H. I. (2007). Biochemical and biophysical characterization of small heat shock proteins from sugarcane. Int. J. Biochem. and Cell Biol. 39, 818–831. 10.1016/j.biocel.2007.01.014 17336576

[B108] Tiroli-Cepeda A. O. Ramos C. H. I. (2010). Heat causes oligomeric disassembly and increases the chaperone activity of small heat shock proteins from sugarcane. Plant Physiology Biochem. 48, 108–116. 10.1016/j.plaphy.2010.01.001 20137963

[B109] Todd O. E. Simpson S. Scheffler B. Dorn K. M. (2024). A fully phased, chromosome-scale genome of sugar beet line FC309 enables the discovery of Fusarium yellows resistance QTL. DNA Res. 32, dsae032. 10.1093/dnares/dsae032 39589284 PMC11747354

[B110] Urnov F. D. Rebar E. J. Holmes M. C. Zhang H. S. Gregory P. D. (2010). Genome editing with engineered zinc finger nucleases. Nat. Rev. Genet. 11, 636–646. 10.1038/nrg2842 20717154

[B111] Wang Y. Stevanato P. Yu L. Zhao H. Sun X. Sun F. (2017). The physiological and metabolic changes in sugar beet seedlings under different levels of salt stress. J. Plant Res. 130, 1079–1093. 10.1007/s10265-017-0964-y 28711996

[B112] Wang D. Wang L. Su W. Ren Y. You C. Zhang C. (2020). A class III *WRKY* transcription factor in sugarcane was involved in biotic and abiotic stress responses. Sci. Rep. 10, 20964. 10.1038/s41598-020-78007-9 33262418 PMC7708483

[B113] Wang Q. Yu G. Chen Z. Han J. Hu Y. Wang K. (2021a). Optimization of protoplast isolation, transformation and its application in sugarcane (*Saccharum spontaneum* L). Crop J. 9, 133–142. 10.1016/j.cj.2020.05.006

[B114] Wang Y. Wang S. Tian Y. Wang Q. Chen S. Li H. (2021b). Functional characterization of a sugar beet *BvbHLH93* transcription factor in salt stress tolerance. IJMS 22, 3669. 10.3390/ijms22073669 PMC8037259 33915978

[B115] Wang X. Guo Y. Wang Y. Peng Y. Zhang H. Zheng J. (2024). ZmHDT103 negatively regulates drought stress tolerance in maize seedlings. Agronomy 14, 134. 10.3390/agronomy14010134

[B116] Wang J. Li X. Wang Y. Lin J. Chen S. Liu H. (2026a). Genetic architecture of sugarcane traits in a polyploid genomics framework. Nature 654, 1–10. 10.1038/s41586-026-10576-7 PMC13293862 42203877

[B117] Wang T. Zhang Q. Xu Q. Zhao H. Li Z. Zhang J. (2026b). Recent advances in the genomics of sugarcane (*saccharum*). JIPB, Jipb, 70362. 10.1111/jipb.70362 42544392

[B118] Wang W. Wang D. Zhao W. Zhang Y. Zeng Z. Hu Y. (2026c). Highly efficient and genotype‐independent genetic transformation System in sugarcane. Plant Biotechnol. J. 24, 2241–2243. 10.1111/pbi.70486 PMC13140336 41348344

[B119] Wang Z. Wang J. Cheng M. Du Y. Godwin I. Wang L. (2026d). Biotechnological and genetic innovations to enhance sorghum adaptation under climate change. Front. Plant Sci. 17, 1757792. 10.3389/fpls.2026.1757792 PMC12967974 41809630

[B120] Wu G.-Q. Li Z.-Q. Cao H. Wang J.-L. (2019a). Genome-wide identification and expression analysis of the *WRKY* genes in sugar beet (*Beta vulgaris* L.) under alkaline stress. PeerJ 7, e7817. 10.7717/peerj.7817 31632850 PMC6796966

[B121] Wu G.-Q. Wang J.-L. Li S.-J. (2019b). Genome-Wide identification of Na^+^/H^+^ antiporter (NHX) genes in sugar beet (*Beta vulgaris* L.) and their regulated expression under salt stress. Genes 10, 401. 10.3390/genes10050401 31137880 PMC6562666

[B122] Wu Q. Pan Y.-B. Su Y. Zou W. Xu F. Sun T. (2022). WGCNA identifies a comprehensive and dynamic gene Co-Expression network that associates with smut resistance in sugarcane. Int. J. Mol. Sci. 23, 10770. 10.3390/ijms231810770 36142681 PMC9506403

[B123] Wu G.-Q. Xie L.-L. Wang J.-L. Wang B.-C. Li Z.-Q. (2023). Genome-Wide identification of CIPK genes in sugar beet (beta vulgaris) and their expression under NaCl stress. J. Plant Growth Regul. 42, 260–274. 10.1007/s00344-021-10545-6

[B124] Wu X. Xia M. Su P. Zhang Y. Tu L. Zhao H. (2024). *MYB* transcription factors in plants: a comprehensive review of their discovery, structure, classification, functional diversity and regulatory mechanism. Int. J. Biol. Macromol. 282, 136652. 10.1016/j.ijbiomac.2024.136652 39427786

[B125] Wu D. Li B. Gu Y. Wang Y. Wang X. Zhao S. (2026a). A transient protoplast expression system for functional gene validation in sugarcane: focus on drought response and immune pathways. New Plant Prot. 3, e70045. 10.1002/npp2.70045

[B126] Wu Z. Hu X. Wang W. Luo Z. Ur Rehman N. Zhao P. (2026b). *ScD27.2* gene regulation mechanism during sugarcane tillering and growth. J. Integr. Agric. 25, 694–708. 10.1016/j.jia.2025.04.017

[B127] Xia J. Zhao Y. Burks P. Pauly M. Brown P. J. (2018). A sorghum *NAC* gene is associated with variation in biomass properties and yield potential. Plant Direct 2, e00070. 10.1002/pld3.70 31245734 PMC6508854

[B128] Xue G. Wu W. Fan Y. Ma C. Xiong R. Bai Q. (2024). Genome-wide identification, evolution, and role of *SPL* gene family in beet (*beta vulgaris* L.) under cold stress. BMC Genomics 25, 101. 10.1186/s12864-024-09995-5 38262939 PMC10804631

[B129] Yang Z. Wang Y. Wei X. Zhao X. Wang B. Sui N. (2017). Transcription profiles of genes related to hormonal regulations under salt stress in sweet sorghum. Plant Mol. Biol. Rep. 35, 586–599. 10.1007/s11105-017-1047-x

[B130] Yang Z. Li J.-L. Liu L.-N. Xie Q. Sui N. (2020). Photosynthetic regulation under salt stress and salt-tolerance mechanism of sweet sorghum. Front. Plant Sci. 10, 1722. 10.3389/fpls.2019.01722 32010174 PMC6974683

[B131] Yang J. Zhang W. Wang T. Xu J. Wang J. Huang J. (2025a). Enhancing sweet sorghum emergence and stress resilience in saline-alkaline soils through ABA seed priming: insights into hormonal and metabolic reprogramming. BMC Genomics 26, 241. 10.1186/s12864-025-11420-4 PMC11905452 40075293

[B132] Yang L. Fang S. Liu L. Zhao L. Chen W. Li X. (2025b). *WRKY* transcription factors: Hubs for regulating plant growth and stress responses. JIPB 67, 488–509. 10.1111/jipb.13828 39815727

[B133] Yanqing W. Jiao L. Lu Z. Hao W. Yiming Z. Irshad A. (2026). Abiotic stress responses in crop plants: a multi-scale approach. J. Integr. Agric. 25, 1–15. 10.1016/j.jia.2024.09.003

[B134] Ye L. Yu J. Zhang X. Yu F. Zeng T. Gu L. (2025). Physiological, transcriptomic and metabolomic analyses reveal that exogenous arginine alleviate the response of *Sorghum bicolor* L. to cadmium stress. Industrial Crops Prod. 229, 120970. 10.1016/j.indcrop.2025.120970

[B135] Yolcu S. Alavilli H. Ganesh P. Asif M. Kumar M. Song K. (2021). An Insight into the abiotic stress responses of cultivated beets (beta vulgaris L.). Plants 11, 12. 10.3390/plants11010012 35009016 PMC8747243

[B136] Zan F. Wu Z. Wang W. Hu X. Feng L. Liu X. (2023). Strigolactones in sugarcane growth and development. Agronomy 13, 1086. 10.3390/agronomy13041086

[B137] Zeng Y. He S. Jing Y. Nong Y. Tang S. Yang Z. (2024). WRKY proteins regulate the development of plants in response to abiotic stresses. J. Plant Interact. 19, 2299865. 10.1080/17429145.2023.2299865

[B138] Zhang Y. Xia P. (2023). The DREB transcription factor, a biomacromolecule, responds to abiotic stress by regulating the expression of stress-related genes. Int. J. Biol. Macromol. 243, 125231. 10.1016/j.ijbiomac.2023.125231 37301338

[B139] Zhang D. Hussain A. Manghwar H. Xie K. Xie S. Zhao S. (2020). Genome editing with the CRISPR-cas system: an art, ethics and global regulatory perspective. Plant Biotechnol. J. 18, 1651–1669. 10.1111/pbi.13383 32271968 PMC7336378

[B140] Zhang H. Zhu J. Gong Z. Zhu J.-K. (2022). Abiotic stress responses in plants. Nat. Rev. Genet. 23, 104–119. 10.1038/s41576-021-00413-0 34561623

[B141] Zhang H.-L. Chen L.-L. Duan Y.-Y. You Z.-Y. Chen J.-S. Bian R.-T. (2024a). Genome-wide analysis of 14-3-3 genes in sugarcane and functional analysis of SsGF2.1 in response to chilling stress. Industrial Crops Prod. 209, 118004. 10.1016/j.indcrop.2023.118004

[B142] Zhang J.-L. Wu G.-Q. Ma B.-T. Wei M. (2024b). Genome-wide identification of the BvHSFs gene family and their expression in sugar beet (*beta vulgaris* L.) under salt stress. Sugar Tech. 26, 1463–1476. 10.1007/s12355-024-01495-w

[B143] Zhang J. Qi Y. Hua X. Wang Y. Wang B. Qi Y. (2025). The highly allo-autopolyploid modern sugarcane genome and very recent allopolyploidization in Saccharum. Nat. Genet. 57, 242–253. 10.1038/s41588-024-02033-w 39753769

[B144] Zhou S.-L. Zhang J.-X. Jiang S. Lu Y. Huang Y.-S. Dong X.-M. (2024). Genome-wide identification of *JAZ* gene family in sugarcane and function analysis of *ScJAZ1/2* in drought stress response and flowering regulation. Plant Physiology Biochem. 210, 108577. 10.1016/j.plaphy.2024.108577 38579542

[B145] Zhou W. Wang Z. G. Li Y. Wu G. J. Li M. Deng Z. L. (2025). Comparative transcriptome and metabolome analysis reveals the differential response to salinity stress of two genotypes brewing sorghum. Sci. Rep. 15, 3365. 10.1038/s41598-025-87100-w 39870699 PMC11772761

[B146] Zhu K. Huang C. Phan T.-T. Yang L.-T. Zhang B.-Q. Xing Y.-X. (2021). Overexpression of SoACLA-1 gene confers drought tolerance improvement in sugarcane. Plant Mol. Biol. Rep. 39, 489–500. 10.1007/s11105-020-01263-6

