# Supplementary material for: CRISPR/Cas genome editing strategies to enhance abiotic stress tolerance in sugar crops
Source: Front Genome Ed. 2026 Oct 14;8:1942811. doi: 10.3389/fgeed.2026.1942811 (PMC13714499; doi:10.3389/fgeed.2026.1942811)
Supplement: Supplementary file 1 [file Table1.doc]

**Table S1:** Chronological milestones in genomic advancement of sugar crops: sugarcane (*Saccharum* spp.), sugar beet (*Beta vulgaris*), and sweet sorghum (*Sorghum bicolor*).

| **Year** | **Milestone** | **Cultivar/Species** | **Genomic Data** | **Key Notes** | **Reference** |
| --- | --- | --- | --- | --- | --- |
| **Sugarcane** | | | | | |
| 2004 | Chloroplast genome sequencing | *S. officinarum* | 141,182 bp | First complete sugarcane chloroplast genome | (Asano, 2004) |
| 2014 | BAC-based reference gene set | R570 (hybrid cultivar) | ~1,400 manually annotated genes | First reference gene set; 317 BACs sequenced; sucrose metabolism pathway analysis | (de Setta et al., 2014) |
| 2018 | Sorghum-referenced haploid assembly | R570 (monoploid) | 382 Mb; 25,316 genes | Mosaic monoploid reference; 17% non-colinear with sorghum | (Garsmeur et al., 2018) |
| 2018 | Whole-genome sequencing | *S. spontaneum* AP85-441 (autotetraploid, 2n=4x=32, x=8) | 2.9 Gb assembled: 35,525 genes | Basic chromosome number reduced from x=10 to x=8; allele-defined genome | (Zhang et al., 2018) |
| 2019 | Synthetic long-read gene-space assembly | SP80-3280 (Brazilian hybrid) | ~3.0 Gb; ~37,000 gene models | 2–6 homo(eo)logs per gene; functional diversity in biomass crop | (Souza et al., 2019) |
| 2022 | PacBio + Hi-C chromosome-scale assembly | Khon Kaen 3 (KK3, Southeast Asian cultivar) | 7.0 Gb; 56 pseudochromosomes; 242,406 genes | First chromosome-scale assembly of commercial cultivar; chimeric assembly due to subgenome similarity | (Shearman et al., 2022) |
| 2022 | Whole-genome sequencing | *S. spontaneum* Np-X (autotetraploid, 2n=4x=40, x=10) | 2.76 Gb; 40 pseudochromosomes; 45,014 genes | Ancestral karyotype (x=10); chromosome reduction to x=8 traced to ~0.8 Mya | (Zhang et al., 2022) |
| 2023 | Whole-genome sequencing | *Erianthus rufipilus* (2 accessions, 2n=2x=20) | 902 Mb / 856.4 Mb; 10 chromosomes; ~33,000 genes | First *Erianthus* genome; closest diploid relative to *Saccharum* | (Kui et al., 2023) |
| 2024 | Haplotype-resolved sequencing | ZZ1 (modern hybrid cultivar) | 10.4 Gb; 114 chromosomes (99 original + 15 recombined); 68,509 genes | Sugar genes from *S. officinarum* subgenome; disease resistance from *S. spontaneum* subgenome | (Bao et al., 2024) |
| 2024 | Polyploid reference genome | R570 (cultivar, ~2n=12x=114) | 8.7 Gb; ~114 chromosomes; 194,593 genes | Resolved *Bru1* brown rust resistance locus; first comprehensive polyploid reference | (Healey et al., 2024) |
| 2025 | Haplotype-resolved sequencing; allo-autopolyploid genome | XTT22 (elite Chinese cultivar) | 9.3 Gb; 97 chromosomes; high-confidence gene set | First haplotype-resolved *allo-autopolyploid* sugarcane genome; recent allopolyploidization event | (Zhang et al., 2025) |
| 2025 | Population & domestication genomics | *S. officinarum*, *S. robustum*, 390 accessions | Whole-genome sequence data; population genome panel | Domestication center confirmed in New Guinea; mosaic genome structure; discovered novel wild *Saccharum* contributor | (Garsmeur et al., 2025) |
| 2026 | Multiscale graph pangenome | 4 *Saccharum* species; 9 genome assemblies | ~14.7 Gb graph; 425.9 million nodes; 47–57 haplotypes per chromosome | First sugarcane graph pangenome; captures ~82% of diversity; dosage-GWAS enabled; *SaTB1* validated via CRISPR | (Huang et al., 2026) |
| 2026 | Fully haplotype-resolved chromosome-scale genome + global population resequencing + polyploid KMERIA-GWAS | POJ2878 (foundational global hybrid cultivar, "King of Sugarcane"); *S. officinarum* XZ & S. *spontaneum* 82-114 (T2T monoploid progenitors); 981 global *Saccharum* accessions | 10.37 Gb final POJ2878 assembly (118 chromosomes; contig N50=15.60 Mb; total annotated 398,858 protein-coding alleles; 7.18 Gb (69.24%) transposon content; 11.43M SNPs from population resequencing) | Pore-C + PacBio HiFi + ONT-UL long-read assembly via self-developed C-Phasing; genome split 80.5% *S.* *officinarum* /19.5% *S.* *spontaneum*, 10 recombinant chromosomes identified; SUS2 favorable *S. spontaneum-*derived haplotype linked to elevated sucrose; *ShSUT2* functionally validated to enlarge stem parenchyma & boost sugar; 95.3% modern cultivars share ≥821.6 Mb IBD with POJ2878; identified domestication/improvement selective sweeps for *CBL1*(cold), *TB1*(tillering), *TIP1*(cell size); custom Allele-Express & KMERIA polyploid bioinformatic tools developed | (Wang et al., 2026) |
| **Sugar beets** | | | | | |
| 2014 | Chloroplast genome sequencing | *B. vulgaris* ssp. | 149,637 bp; 113 genes (79 CDS, 30 tRNA, 4 rRNA) | First complete chloroplast genome (GenBank KJ081864) | (Li et al., 2014) |
| 2014 | First nuclear reference genome | KWS2320 (doubled haploid) | ~731 Mb; 27,421 protein-coding genes; 9 pseudochromosomes | First non-rosid/asterid eudicot reference; RefBeet-1.2 | (Dohm et al., 2014) |
| 2015 | SMRT-based chloroplast assembly | *B. vulgaris* ssp. | 149,637 bp; resolved inverted repeats | Long-read assembly from nuclear sequencing data; outperformed short-read assemblies | (Stadermann et al., 2015) |
| 2019 | Wild beet genome draft | *B. vulgaris* ssp. *maritima* (sea beet) | ~730 Mb; 27,662 genes | Wild relative of cultivated sugar beet; important for trait introgression | (Rodríguez Del Río et al., 2019) |
| 2021 | Chard genome assembly | *B. vulgaris* ssp. *var. cicla* (chard) | 604 Mb; 34,521 genes; ~57% repetitive sequence | First chard genome; synteny with sugar beet; haplotype sharing | (Lehner et al., 2021) |
| 2022 | Chromosome-scale assembly | IMA1 (pure line) | 786 Mb; 9 pseudochromosomes; 35,003 genes; N50 = 367.5 kb | PacBio + Hi-C; GWAS identified 10 disease-resistance genes | (Li et al., 2022) |
| 2022 | Pan-plastome sequencing | 11 *Beta* / *Patellifolia* species | 149.7–152.8 kb | High-resolution phylogeny of beet wild relatives; resolved *Beta* sections *Beta* and *Corollinae* | (Sielemann et al., 2022) |
| 2023 | Contiguous de novo genome assembly | EL10 (sugar beet inbred line) | 540 Mb (EL10.1); 24,255 gene models; 96.7% in 9 pseudochromosomes | PacBio + BioNano + Hi-C; reduced gene copy number vs. other eudicots | (McGrath et al., 2023) |
| 2023 | Pangenome of cultivated beet and CWRs | *B. corolliflora*, *B. lomatogona*, *B. macrorhiza*, *P. procumbens* | Comprehensive pangenome dataset | Parental relationships: *B. corolliflora* is autotetraploid from *B. macrorhiza* | (Sielemann et al., 2023) |
| 2023 | GWAS of 13 agronomic traits | 977 sugar beet accessions | 170,750 SNPs (GBS) | Candidate genes for taproot traits (RGD, RS, CS, FC) and aboveground traits | (Liu et al., 2023) |
| 2024 | Repeatome evolution and speciation | 13 beet species (all sections of Beta and Patellifolia) | Genome sizes and repeat profiles; satellite DNA variability | Repetitive DNAs causal for genome expansions/contractions; distinct centromere architectures | (Schmidt et al., 2024) |
| 2024 | GWAS of biomass and sugar content | 207 sugar beet accessions | 14.6 million SNPs; 15× depth | Identified UDP-glucose 4-epimerase balancing sugar accumulation and cell wall synthesis | (Wang et al., 2024) |
| 2024 | Domestication selection signatures | *Beta* diversity panel (wild + cultivated) | 10.3 million SNP markers; 4 genetic clusters | Candidate genes for root development, lateral root suppression, sucrose metabolism | (Dhiman et al., 2025) |
| 2025 | Long-read nuclear upgrade | KWS2320 (doubled haploid) | 648 Mb; N50 = 61.5 Mb; 28,271 genes (RefBeet-3.0, BeetSet-3) | Chromosome-scale with PacBio long reads | (Dohm et al., 2025) |
| **Sweet sorghum** | | | | | |
| 2009 | First reference genome | *S. bicolor* BTx623 (grain sorghum) | ~730 Mb; ~34,000 genes | First published sorghum genome; serves as reference for sweet sorghum studies | (Paterson et al., 2009) |
| 2011 | Genome resequencing - sweet vs. grain | Keller (sweet), BTx623, IS3620C (grain) | Comparative genomics | Identified SNPs, indels, and SVs differentiating sweet from grain sorghum | (Zheng et al., 2011) |
| 2018 | Improved reference assembly | *S. bicolor* (BTx623 v3.1) | Improved contiguity; transcriptome atlas | First high-quality reference with 10 pseudochromosomes; tissue-specific expression atlas | (McCormick et al., 2018) |
| 2024 | Sweet sorghum super transcriptome | 7-sweet sorghum cultivars | 45,864 RTAs; 2,802 new genes added to SSRG | Identified 58 sweet sorghum-exclusive genes: improved genome annotation via RNA-seq | (Nikhil et al., 2024) |
| 2025 | GWAS and haplotype analysis | 183-sweet sorghum accessions | 14,819 high-quality SNP markers; 21 QTNs identified | Four candidate genes: threonine protein kinase (flowering), ethylene-insensitive (Brix), protein kinase & amino acid transporter (biomass) | (Umar et al., 2025) |
| 2025 | Telomere-to-telomere genome assembly & multi-omics resource | E048 (inbred sorghum line) | 729.46 Mb complete genome; expression atlas (13 tissues); 13,226 mutant libraries | SGMD database; 2,291,074 mutations covering 97.54% of genes; *Agrobacterium* transformation platform | (Chen et al., 2025) |

Table S1: summarizes landmark genomic achievements, including organelle sequencing, chromosome-scale and haplotype-resolved assemblies, pangenome construction, population genomic analyses, and functional characterization via., GWAS, and gene editing. Entries are organized by year, crop, and key methodological or biological advances, highlighting improvements in assembly quality, ploidy resolution, evolutionary insights, and trait-associated gene discovery, biomass production, and stress resistance.

**References**

Asano, T. (2004). Complete Nucleotide Sequence of the Sugarcane (*Saccharum Officinarum*) Chloroplast Genome: A Comparative Analysis of Four Monocot Chloroplast Genomes. *DNA Research* 11, 93–99. doi: 10.1093/dnares/11.2.93

Bao, Y., Zhang, Q., Huang, J., Zhang, S., Yao, W., Yu, Z., et al. (2024). A chromosomal-scale genome assembly of modern cultivated hybrid sugarcane provides insights into origination and evolution. *Nat Commun* 15, 3041. doi: 10.1038/s41467-024-47390-6

Chen, C., Ge, F., Du, H., Sun, Y., Sui, Y., Tang, S., et al. (2025). A comprehensive omics resource and genetic tools for functional genomics research and genetic improvement of sorghum. *Molecular Plant* 18, 703–719. doi: 10.1016/j.molp.2025.03.005

de Setta, N., Monteiro-Vitorello, C. B., Metcalfe, C. J., Cruz, G. M. Q., Del Bem, L. E., Vicentini, R., et al. (2014). Building the sugarcane genome for biotechnology and identifying evolutionary trends. *BMC Genomics* 15, 540. doi: 10.1186/1471-2164-15-540

Dhiman, A. S., Taliadoros, D., Stukenbrock, E. H., McGrath, J. M., Emrani, N., and Jung, C. (2025). Signatures in domesticated beet genomes pointing at genes under selection in a sucrose-storing root crop. *BMC Biol* 23, 299. doi: 10.1186/s12915-025-02422-5

Dohm, J. C., Holzweber, T., Pensch, R. A., and Himmelbauer, H. (2025). Sugar beet long-read reference assembly of genotype KWS2320. *NAR Genom Bioinform* 7, lqaf142. doi: 10.1093/nargab/lqaf142

Dohm, J. C., Minoche, A. E., Holtgräwe, D., Capella-Gutiérrez, S., Zakrzewski, F., Tafer, H., et al. (2014). The genome of the recently domesticated crop plant sugar beet (*Beta vulgaris*). *Nature* 505, 546–549. doi: 10.1038/nature12817

Garsmeur, O., Droc, G., Antonise, R., Grimwood, J., Potier, B., Aitken, K., et al. (2018). A mosaic monoploid reference sequence for the highly complex genome of sugarcane. *Nat Commun* 9, 2638. doi: 10.1038/s41467-018-05051-5

Garsmeur, O., Rio, S., Pompidor, N., Lipzen, A., Hervouet, C., Durand, T., et al. (2025). The genomic footprints of wild *Saccharum* species trace domestication, diversification, and modern breeding of sugarcane. *Cell* 188, 7252-7266.e15. doi: 10.1016/j.cell.2025.09.017

Healey, A. L., Garsmeur, O., Lovell, J. T., Shengquiang, S., Sreedasyam, A., Jenkins, J., et al. (2024). The complex polyploid genome architecture of sugarcane. *Nature*, 1–7. doi: 10.1038/s41586-024-07231-4

Huang, Y., Zhang, Y., Zhang, Q., Zhuang, G., Li, C., Wang, B., et al. (2026). Multiscale pangenome graphs empower the genomic dissection of mixed-ploidy sugarcane species. *Science* 391, eadx1616. doi: 10.1126/science.adx1616

Kui, L., Majeed, A., Wang, X., Yang, Z., Chen, J., He, L., et al. (2023). A chromosome-level genome assembly for *Erianthus fulvus* provides insights into its biofuel potential and facilitates breeding for improvement of sugarcane. *Plant Commun* 4, 100562. doi: 10.1016/j.xplc.2023.100562

Lehner, R., Blazek, L., Minoche, A. E., Dohm, J. C., and Himmelbauer, H. (2021). Assembly and characterization of the genome of chard (Beta vulgaris ssp. vulgaris var. cicla). *Journal of Biotechnology* 333, 67–76. doi: 10.1016/j.jbiotec.2021.04.007

Li, H., Cao, H., Cai, Y.-F., Wang, J.-H., Qu, S.-P., and Huang, X.-Q. (2014). The complete chloroplast genome sequence of sugar beet (*Beta vulgaris* ssp. *vulgaris* ). *Mitochondrial DNA* 25, 209–211. doi: 10.3109/19401736.2014.883611

Li, X., He, W., Fang, J., Liang, Y., Zhang, H., Chen, D., et al. (2022). Genomic and transcriptomic-based analysis of agronomic traits in sugar beet (*Beta vulgaris* L.) pure line IMA1. *Front. Plant Sci.* 13, 1028885. doi: 10.3389/fpls.2022.1028885

Liu, D., Tan, W., Wang, H., Li, W., Fu, J., Li, J., et al. (2023). Genetic diversity and genome-wide association study of 13 agronomic traits in 977 *Beta vulgaris* L. germplasms. *BMC Genomics* 24, 413. doi: 10.1186/s12864-023-09522-y

McCormick, R. F., Truong, S. K., Sreedasyam, A., Jenkins, J., Shu, S., Sims, D., et al. (2018). The *Sorghum bicolor* reference genome: improved assembly, gene annotations, a transcriptome atlas, and signatures of genome organization. *The Plant Journal* 93, 338–354. doi: 10.1111/tpj.13781

McGrath, J. M., Funk, A., Galewski, P., Ou, S., Townsend, B., Davenport, K., et al. (2023). A contiguous *de novo* genome assembly of sugar beet EL10 (*Beta vulgaris* L.). *DNA Research* 30, dsac033. doi: 10.1093/dnares/dsac033

Nikhil, S., Mohideen, H. S., and Sella, R. N. (2024). Unveiling the Genomic Symphony: Identification Cultivar-Specific Genes and Enhanced Insights on Sweet Sorghum Genomes Through Comprehensive superTranscriptomic Analysis. *J Mol Evol* 92, 720–743. doi: 10.1007/s00239-024-10198-5

Paterson, A. H., Bowers, J. E., Bruggmann, R., Dubchak, I., Grimwood, J., Gundlach, H., et al. (2009). The *Sorghum bicolor* genome and the diversification of grasses. *Nature* 457, 551–556. doi: 10.1038/nature07723

Rodríguez Del Río, Á., Minoche, A. E., Zwickl, N. F., Friedrich, A., Liedtke, S., Schmidt, T., et al. (2019). Genomes of the wild beets *Beta patula* and *Beta vulgaris* ssp. *maritima*. *The Plant Journal* 99, 1242–1253. doi: 10.1111/tpj.14413

Schmidt, N., Sielemann, K., Breitenbach, S., Fuchs, J., Pucker, B., Weisshaar, B., et al. (2024). Repeat turnover meets stable chromosomes: repetitive DNA sequences mark speciation and gene pool boundaries in sugar beet and wild beets. *The Plant Journal* 118, 171–190. doi: 10.1111/tpj.16599

Shearman, J. R., Pootakham, W., Sonthirod, C., Naktang, C., Yoocha, T., Sangsrakru, D., et al. (2022). A draft chromosome-scale genome assembly of a commercial sugarcane. *Sci Rep* 12, 20474. doi: 10.1038/s41598-022-24823-0

Sielemann, K., Pucker, B., Schmidt, N., Viehöver, P., Weisshaar, B., Heitkam, T., et al. (2022). Complete pan-plastome sequences enable high resolution phylogenetic classification of sugar beet and closely related crop wild relatives. *BMC Genomics* 23, 113. doi: 10.1186/s12864-022-08336-8

Sielemann, K., Schmidt, N., Guzik, J., Kalina, N., Pucker, B., Viehöver, P., et al. (2023). Pangenome of cultivated beet and crop wild relatives reveals parental relationships of a tetraploid wild beet. doi: 10.1101/2023.06.28.546919

Souza, G. M., Van Sluys, M.-A., Lembke, C. G., Lee, H., Margarido, G. R. A., Hotta, C. T., et al. (2019). Assembly of the 373k gene space of the polyploid sugarcane genome reveals reservoirs of functional diversity in the world’s leading biomass crop. *Gigascience* 8, giz129. doi: 10.1093/gigascience/giz129

Stadermann, K. B., Weisshaar, B., and Holtgräwe, D. (2015). SMRT sequencing only de novo assembly of the sugar beet (*Beta vulgaris*) chloroplast genome. *BMC Bioinformatics* 16, 295. doi: 10.1186/s12859-015-0726-6

Umar, A. B., Ramalingam, A. P., Sadia, B., Awan, F. S., Khan, F. A., Nasir, M., et al. (2025). Genome-wide association study and haplotype analyses reveal the genetic architecture of agronomic traits and sugars in sweet sorghum. *Front. Genet.* 16, 1611863. doi: 10.3389/fgene.2025.1611863

Wang, J., Li, X., Wang, Y., Lin, J., Chen, S., Liu, H., et al. (2026). Genetic architecture of sugarcane traits in a polyploid genomics framework. *Nature*, 1–10. doi: 10.1038/s41586-026-10576-7

Wang, S., Yue, Z., Yu, C., Wang, R., Sui, Y., Hou, Y., et al. (2024). Genome-wide association study identifies the genetic basis of key agronomic traits in 207 sugar beet accessions. *Horticulture Research* 11, uhae230. doi: 10.1093/hr/uhae230

Zhang, J., Qi, Y., Hua, X., Wang, Y., Wang, B., Qi, Y., et al. (2025). The highly allo-autopolyploid modern sugarcane genome and very recent allopolyploidization in *Saccharum*. *Nat Genet* 57, 242–253. doi: 10.1038/s41588-024-02033-w

Zhang, J., Zhang, X., Tang, H., Zhang, Q., Hua, X., Ma, X., et al. (2018). Allele-defined genome of the autopolyploid sugarcane *Saccharum spontaneum* L. *Nat Genet* 50, 1565–1573. doi: 10.1038/s41588-018-0237-2

Zhang, Q., Qi, Y., Pan, H., Tang, H., Wang, G., Hua, X., et al. (2022). Genomic insights into the recent chromosome reduction of autopolyploid sugarcane *Saccharum spontaneum*. *Nat Genet* 54, 885–896. doi: 10.1038/s41588-022-01084-1

Zheng, L.-Y., Guo, X.-S., He, B., Sun, L.-J., Peng, Y., Dong, S.-S., et al. (2011). Genome-wide patterns of genetic variation in sweet and grain sorghum (*Sorghum bicolor*). *Genome Biol* 12, R114. doi: 10.1186/gb-2011-12-11-r114
